# Moderators of Sexual Recidivism as Indicator of Treatment Effectiveness in Persons With Sexual Offense Histories: An Updated Meta-analysis

**DOI:** 10.1177/10790632231159071

**Published:** 2023-03-16

**Authors:** Lisa Holper, Andreas Mokros, Elmar Habermeyer

**Affiliations:** 1 Department of Forensic Psychiatry, 27217 University Hospital of Psychiatry Zurich, Zurich, Switzerland; 2 Department of Psychology, 26553 FernUniversität in Hagen, Hagen, Germany

**Keywords:** sexual offending, sexual offense treatment, treatment predictors, risk assessment, heterogeneity

## Abstract

The present meta-analysis is an update of the meta-analysis by Schmucker and Lösel [Campbell Syst. Rev. 2017; 13: 1–75], which synthesized evidence on sexual recidivism as an indicator of treatment effectiveness in persons with sexual offense histories. The updated meta-analysis includes 37 samples comprising a total of 30,394 individuals with sexual offense histories, which is nearly three times the sample size reported by Schmucker and Lösel (2017: 28 samples, *N* = 9781). In line with Schmucker and Lösel (2017), the mean treatment effect was small with an odds ratio of 1.54 [95% CI 1.22, 1.95] (*p* < .001). A moderator analysis suggested three predictors of importance, i.e., risk level, treatment specialization, and author confounding. Greater treatment effectiveness was suggested in high- and medium-compared to low-risk individuals and in specialized compared to non-specialized treatments. Authors affiliated with treatment programs reported larger effectiveness than independent authors. These findings were overall in line with Schmucker and Lösel (2017), though the effects of risk level and treatment specialization were stronger in the current meta-analysis. The findings of the updated meta-analysis reinforce the evidence for the first and second principle of the Risk-Need-Responsivity model. The results may support researchers and decision-makers in interpreting the current evidence on sexual recidivism as an indicator of treatment effectiveness, and, based on that, implement and carry out informative, methodologically sound evaluations of ongoing treatment programs in persons with sexual offense histories.

## Introduction

Treatment of persons who committed sexual offenses focuses on the reduction of sexual recidivism in order to increase public safety. Although previous meta-analyses provided evidence for reductions in sexual recidivism (Alexander, 1999; Aos et al., 2006; Furby et al., 1989; Gallagher et al., 1999; Gannon et al., 2019; Grossman et al., 1999; Hall, 1995; Hanson et al., 2002, 2009a, 2009b; Kim et al., 2016; Lösel & Schmucker, 2005; Mpofu et al., 2018; Polizzi et al., 1999; Reitzel & Carbonell, 2006; Schmucker & Lösel, 2015, 2017; Ter Beek et al., 2018; Walker et al., 2004), there is still controversy about which sample characteristics, treatment variables, or methodological issues contribute most to the effectiveness of treatment (Lösel, 2020). Sound treatment evaluation in this field is complicated by various concerns, such as the heterogeneity among persons with sexual offense histories in terms of pre-treatment risk of reoffending, variances between adults and juveniles, differences in treatment approaches, and deficits in study design assigning appropriate treatment and control groups (Lösel & Schmucker, 2017).

In 2005, Lösel and Schmucker (2005) found that interventions that incorporated behavior therapy significantly reduced sexual recidivism. More recently, Gannon et al. (2019) reported that interventions that incorporated behavior therapy produced larger reductions in sexual recidivism than those that did not. These findings should, however, be interpreted with caution because the studies of treatment programs included by Lösel and Schmucker (2005) and by Gannon et al. (2019) were heavily confounded because of the inclusion of studies with weak study designs. In a subsequent meta-analysis using more rigorous eligibility criteria, Schmucker and Lösel (2017) therefore excluded a vast proportion of these studies. Based on this evidence, the meta-analysis by Schmucker and Lösel (2017) may be regarded as the methodologically most convincing synthesis on treatment effectiveness in persons with sexual offense histories as indicated by sexual recidivism. Schmucker and Lösel (2017) mentioned that a further update was planned in about 2 years’ time to include more recent evaluations, which has not been published so far. The present work therefore aimed to update their 2017 meta-analysis.

Schmucker and Lösel (2017) applied rigorous eligibility criteria based on the Maryland Scientific Methods Scale (SMS) (Farrington et al., 2002). The SMS is a five-point scale ranging from level 1, for evaluations based on simple cross sectional correlations, to level 5, for randomized control trials. In order to be included, all studies within the Schmucker and Lösel (2017) meta-analysis had to fulfill at least level 3 (incidental assignment), level 4 (matching procedures), or level 5 (randomized controlled trial, RCT) to ensure equivalence between treatment and control groups. All studies had to compare official sexual recidivism rates of treated persons with sexual offense histories with a control group that had not been subjected to the respective treatment. All studies had to explicitly aim at reducing sexual recidivism, even though treatments were not required to be specialized for sexual offending. Both adult and juvenile samples were considered. Based on these eligibility criteria, Schmucker and Lösel (2017) included 27 studies (Bakker et al., 1998; Borduin et al., 1990, 2009; Duwe & Goldman, 2009; Friendship et al., 2003; Greenberg et al., 2002; Guarino-Ghezzi & Kimball, 1998; Hanson et al., 1992, 2004; Lab et al., 1993; LaMacaza, 2002; Looman et al., 2000; Marques et al., 2005; Marshall et al., 1991; Marshall & Barbaree, 1988; McGrath et al., 1998; Nicholaichuk, 1996; Ortmann, 2002; Procter, 1996; Rice et al., 1991; Romero & Williams, 1983; Ruddijs & Timmerman, 2000; Schmid, 1989; Taylor, 2000; Worling & Curwen, 2000; Ziethen, 2002) published between 1983 and 2009. Overall, there was a statistically significant mean treatment effect for sexual reoffending with an odds ratio of *OR* 1.41 [95% CI 1.11, 1.78] (*p* = .005) equating to 26.4% less sexual recidivism after treatment (mean n-weighted sexual recidivism rate of 10.1% in treated vs. 13.7% in untreated groups). This effect size is small considering the equivalent to Cohen’s d (Cohen’s *d* > .2) (Chen et al., 2010; Cohen, 1988). This relatively low treatment efficacy is in line with findings from the Sex Offender Treatment Programme (SOTP) (Dennis et al., 2012; Völlm, 2018) and is not unique to sexual recidivism but has also been reported with respect to general recidivism (Beaudry et al., 2021), for example. Depending on the evaluation design even negative effects, thus recidivism-promoting effects, have been observed in the SOTP (Lösel et al., 2020; Mews et al., 2017).

Schmucker and Lösel (2017) also conducted a moderator analysis, which suggested several factors to be significantly associated with treatment effectiveness. The strongest moderating effect was observed for risk level, i.e., the pre-treatment risk of reoffending, suggesting that treatment programs focusing on high- or medium-risk compared to low-risk individuals lead to greater reduction in sexual recidivism. Another strong moderating effect resulted for descriptive validity, i.e., the quality of study reporting, suggesting that unsatisfactory reports went along with worse treatment outcomes. In addition, programs applying treatment approaches based on cognitive-behavioral therapy (CBT) or multisystemic therapy (MST) in juveniles showed modest but significant effects on sexual recidivism, though the difference to other psychotherapeutic approaches did not reach statistical significance. Further, programs carrying out more individualized compared to group-based treatment were related to greater treatment effectiveness. Specialized versus non-specialized treatments, however, did not differ in effectiveness. At last, there was a small-study effect suggesting that larger samples yielded slightly worse treatment effectiveness, a phenomenon commonly observed in meta-analyses (Hong et al., 2020).

Overall, these observations supported the first principle of the Risk-Need-Responsivity (RNR) model, which outlines that treatment allocation should be guided by individuals’ risk levels, to generate effective interventions (Bonta & Andrews, 2007). The second and third principles, stating that treatment should be specific to the individuals’ criminogenic needs and be delivered attuned to their learning and motivational style, were not sufficiently evidenced (Hanson et al., 2009a, 2009b). Although Schmucker and Lösel (2017) suggested these findings to be promising, the large residual heterogeneity observed did not allow to draw general conclusions about the effectiveness of treatment in persons with sexual offense histories.

The aim of the present analysis was consequently to update the meta-analysis by Schmucker and Lösel (2017) in order to evaluate whether more recent studies that might have been published in the field after the completion of their meta-analysis, would provide more robust evidence on the factors moderating treatment effectiveness in persons with sexual offense histories.

## Methods

The following sections report how studies were selected, how sample size was determined, and all data exclusions. The authors take responsibility for the integrity of the data, the accuracy of the data analyses, and have made every effort to avoid inflating statistically significant results. Research ethics approval was not applicable.

### Study Selection

Databases including the Center for Sex Offender Management (CSOM) documents database, Cochrane Library, Dissertation Abstracts International, MedLine, ProQuest Dissertations & Theses Database, PsycInfo, and Psyndex were searched using the Boolean terms sex AND treat* or sex AND therap* together with the terms recidivi* OR reoffend*. The time frame considered was primarily from 2009 until 2022, since the meta-analysis by Schmucker and Lösel (2017) included studies up to 2009. In addition, we also searched other meta-analyses in the field for studies that might have been eligible but not included in their meta-analysis (Alexander, 1999; Aos et al., 2006; Furby et al., 1989; Gallagher et al., 1999; Gannon et al., 2019; Grossman et al., 1999; Hall, 1995; Hanson et al., 2002, 2009a, 2009b; Kim et al., 2016; Lösel & Schmucker, 2005; Mpofu et al., 2018; Polizzi et al., 1999; Reitzel & Carbonell, 2006; Schmucker & Lösel, 2015; Ter Beek et al., 2018; Walker et al., 2004). The reason why Schmucker and Lösel (2017) considered only studies up to 2009 although their meta-analysis was published in 2017, is unknown to the current authors.

Following Schmucker and Lösel (2017), eligible studies had to (1) include males irrespective of age, (2) contain a minimum sample size of ten subjects, (3) fulfill at least level 3 study design on the SMS to ensure equivalence between treatment and control groups, (4) provide official recidivism rates with respect to sexual recidivism, and (5) the treatment approach had to explicitly aim at reducing sexual recidivism rates. There were no restrictions regarding the country of origin in which studies were conducted or whether studies were published or unpublished.

The updated meta-analysis was based on the 27 primary studies identified by Schmucker and Lösel (2017) (Bakker et al., 1998; Borduin et al., 1990, 2009; Duwe & Goldman, 2009; Friendship et al., 2003; Greenberg et al., 2002; Guarino-Ghezzi & Kimball, 1998; Hanson et al., 1992, 2004; Lab et al., 1993; LaMacaza, 2002; Looman et al., 2000; Marques et al., 2005; Marshall et al., 1991; Marshall & Barbaree, 1988; McGrath et al., 1998; Nicholaichuk, 1996; Ortmann, 2002; Procter, 1996; Rice et al., 1991; Romero & Williams, 1983; Ruddijs & Timmerman, 2000; Schmid, 1989; Taylor, 2000; Worling & Curwen, 2000; Ziethen, 2002). Upon the search, two of the original studies were updated with more recent publications on the same samples, one (Worling et al., 2010) recommended in the Online Supplementary Materials by Schmucker and Lösel (2017), the other (Borduin et al., 2021) identified by the current authors. Another six studies (Abracen et al., 2011; Grady et al., 2017; Letourneau et al., 2013; Olver et al., 2020; Smallbone & McHugh, 2010; Smid et al., 2016) recommended in the Online Supplementary Materials by Schmucker and Lösel (2017) as being eligible for updating their meta-analysis, were also added; again, two of these recommended studies were updated with more recent studies on the same samples (Grady et al., 2017; Olver et al., 2020) identified by the current authors. Another two eligible studies (Buttars et al., 2016; Mews et al., 2017) were identified based on other recent meta-analyses (Gannon et al., 2019; Lösel, 2020).

Following Schmucker and Lösel (2017), if studies reported statistical analyses controlling for differences between treatment and control groups (e.g., regression methods including relevant control variables), the resulting adjusted recidivism rates were used instead of raw recidivism rates. If studies reported information on dropouts, those were included in the treatment groups according to an intention-to-treat analysis. If studies reported multiple treatment and/or control groups, the comparison with the highest internal validity was used. If studies reported recidivism rates for a matched subsample of treatment and control groups on relevant characteristics, this was used instead of the total sample. If studies reported separate recidivism rates for different offender types or risk groups (Greenberg et al., 2002; Marshall & Barbaree, 1988), these were reported separately. A PRISMA flow chart illustrating the study selection process is provided in the Online Supplementary Materials.

## Outcomes

The final data set included in the updated meta-analysis consisted of 35 studies (Abracen et al., 2011; Bakker et al., 1998; Borduin et al., 1990, 2021; Buttars et al., 2016; Duwe & Goldman, 2009; Friendship et al., 2003; Grady et al., 2017; Greenberg et al., 2002; Guarino-Ghezzi & Kimball, 1998; Hanson et al., 1992, 2004; Lab et al., 1993; LaMacaza, 2002; Letourneau et al., 2013; Looman et al., 2000; Marques et al., 2005; Marshall et al., 1991; Marshall & Barbaree, 1988; McGrath et al., 1998; Mews et al., 2017; Nicholaichuk, 1996; Olver et al., 2020; Ortmann, 2002; Procter, 1996; Rice et al., 1991; Robinson, 1995; Romero & Williams, 1983; Ruddijs & Timmerman, 2000; Schmid, 1989; Smallbone & McHugh, 2010; Smid et al., 2016; Taylor, 2000; Worling et al., 2010; Ziethen, 2002). The main characteristics of the studies included are listed in Table 1.

**Table 1.** Updated meta-analysis. Main characteristics of the 35 studies included in the updated meta-analysis. Listed are country of origin, treatment approach, treatment specialization, design quality, risk level, and age group.

Study	Country	Approach	Specialization	Design	Risk Level	Age
Abracen et al. 2011	Canada	Cognitive-behavioral therapy	Yes	Level 4	Medium	Adults
Bakker et al. 1998	Other	Cognitive-behavioral therapy	Yes	Level 3	Medium	Adults
Borduin et al. 1990	United States	Multisystemic therapy	Yes	Level 5	High	Juveniles
Borduin et al. 2021	United States	Multisystemic therapy	Yes	Level 5	High	Juveniles
Buttars et al. 2016	United States	Therapeutic community	Yes	Level 4	Medium	Adults
Duwe & Goldman, 2009	United States	Cognitive-behavioral therapy	Yes	Level 4	Medium	Adults
Friendship et al. 2003	Other	Cognitive-behavioral therapy	Yes	Level 4	Medium	Adults
Grady et al. 2017	United States	Cognitive-behavioral therapy	Yes	Level 4	Low	Adults
Greenberg et al. 2002	Other	Cognitive-behavioral therapy	Yes	Level 3	Low	Adults
Guarino-Ghezzi & Kimball, 1998	United States	Cognitive-behavioral therapy	Yes	Level 3	High	Juveniles
Hanson et al. 1992	Canada	Behavioral therapy	Yes	Level 4	Medium	Adults
Hanson et al. 2004	Canada	Insight-oriented therapy	Yes	Level 3	Low	Adults
LaMacaza, 2002	Canada	Cognitive-behavioral therapy	Yes	Level 3	Medium	Adults
Lab et al. 1993	United States	Cognitive-behavioral therapy	Yes	Level 3	Medium	Juveniles
Letourneau et al. 2013	United States	Multisystemic therapy	Yes	Level 5	Medium	Juveniles
Looman et al. 2000	Canada	Behavioral therapy	Yes	Level 4	High	Adults
Marques et al. 2005	United States	Cognitive-behavioral therapy	Yes	Level 5	Medium	Adults
Marshall & Barbaree, 1988	Canada	Behavioral therapy	Yes	Level 3	Medium	Adults
Marshall et al. 1991	Canada	Behavioral therapy	Yes	Level 3	Unspecified	Adults
McGrath et al. 1998	United States	Cognitive-behavioral therapy	Yes	Level 3	High	Adults
Mews et al. 2017	Other	Cognitive-behavioral therapy	Yes	Level 4	Low	Adults
Nicholaichuk, 1996	Canada	Cognitive-behavioral therapy	No	Level 4	Unspecified	Adults
Olver et al. 2020	Canada	Cognitive-behavioral therapy	Yes	Level 3	Medium	Adults
Ortmann, 2002	Other	Therapeutic community	Yes	Level 5	Unspecified	Adults
Procter, 1996	Other	Cognitive-behavioral therapy	Yes	Level 4	Medium	Adults
Rice et al., 1991	Canada	Behavioral therapy	Yes	Level 4	Low	Adults
Robinson, 1995	Canada	Cognitive-behavioral therapy	Yes	Level 5	Unspecified	Adults
Romero & Williams, 1983	United States	Insight-oriented therapy	No	Level 5	Low	Adults
Ruddijs & Timmerman, 2000	Other	Cognitive-behavioral therapy	No	Level 4	Low	Adults
Schmid, 1989	Other	Therapeutic community	Yes	Level 3	High	Adults
Smallbone & McHugh, 2010	Other	Cognitive-behavioral therapy	Yes	Level 3	Medium	Adults
Smid et al. 2016	Other	Cognitive-behavioral therapy	Yes	Level 3	Medium	Adults
Taylor, 2000	Other	Therapeutic community	Yes	Level 3	Unspecified	Adults
Worling et al. 2010	Canada	Cognitive-behavioral therapy	Yes	Level 3	High	Juveniles
Ziethen, 2002	Other	Therapeutic community	Yes	Level 4	Medium	Adults

Primary outcome was the sexual recidivism rate. Schmucker and Lösel (2017) reported that all but one study (Robinson, 1995) provided information on sexual recidivism. The current authors identified the missing information in that study (Table 13 in the corresponding publication, Robinson, 1995). Consequently, the updated meta-analysis collected 37 unique samples from 35 studies, whereas Schmucker and Lösel (2017) had collected 28 samples from 26 studies with respect to sexual recidivism.

Secondary outcomes were violent and general recidivism rates, which were reported in 54% and 65% of the studies, respectively. Because of the small number of studies reporting on violent or general recidivism, an adequate integration of these outcomes was not considered meaningful in the present analysis. Details on these outcomes are therefore only provided in the online supplemental appendix.

### Meta-Analysis

Random-effects meta-analysis was conducted using the rma.mv command in the metafor package (Viechtbauer, 2021) in the R programming language (R Core Team, 2022), which provides a comprehensive collection of functions for fitting meta-analytic models. Sample-specific effect sizes were computed based on the confusion matrices collected in the primary studies using the escalc command. If any of the frequencies equaled zero, .5 was added to each frequency. The analyses were conducted on logged odds ratios and then reported as odds ratio with 95% confidence intervals (OR [95% CI]).

To estimate the expected range of true effects in future similar studies, the 95% prediction interval ([95% PI]) around the mean treatment effect was computed (Borenstein et al., 2021). A PI represents the interval in which future observations will likely fall with a certain probability based on known evidence. A PI accounts for both uncertainty in estimating the population mean plus the variation in individual values. A PI is therefore always wider than a CI.

To check the agreement between the sample-specific effect sizes collected in the present work and that reported by Schmucker and Lösel (2017), the intraclass correlation coefficient (ICC) was computed using a two-way random-effects model and single-rater unit ( $ICC_{\left(A,1\right)}$ ).

To compared the mean treatment effect observed in the updated meta-analysis and that reported by Schmucker and Lösel (2017), a fixed-effects meta-regression model was applied (Viechtbauer, 2021).

Heterogeneity was reported in terms of residual heterogeneity ( Q ) and $I^{2}$ (Higgins & Thompson, 2002).

### Moderator Analysis

Moderator analysis was carried out under the assumption of a mixed-effects model using the rma.mv command in the metafor package (Viechtbauer, 2021). The model was fitted for each moderator separately.

Categorical moderators were reported in terms of subgroup-specific effect sizes (OR [95% CI]). The corresponding subgroup-contrasts were assessed based on general linear hypothesis (GLH) testing using the glht command in the multcomp package (Hothorn et al., 2022) and reported in terms of *z*- and *p*-values. The Bonferroni correction was applied to counteract the problem of multiple comparisons using the p-adjusted option in the glht command, where the *p*-values are multiplied by the number of comparisons.

Continuous moderators were centered, by subtracting the mean, and scaled, by dividing the centered variable by its standard deviation, and reported in terms of regression weights ( β ) and *z*-values following Schmucker and Lösel (2017).

Following the coding scheme provided by Schmucker and Lösel (2017), a total of 17 publication-, sample-, treatment-, and individual-specific moderators were collected (15 categorical predictors, nine continuous predictors). Subgroups of the categorical moderators are listed below in brackets. Details on the coding scheme are provided in the Online Supplementary Materials. The data were coded by one author, it was therefore not possible to provide a measure of inter-rater reliability.

• Publication characteristics (5 moderators): publication status [published, unpublished], publication year [< 2000, ≥ 2000], country [Canada, United States, Other], author confounding [Yes, No, Unclear], descriptive validity.
• Sample characteristics (5 moderators): sample size [< 50, 51–150, 151–250, 251–500, >500], design quality [Level 3 (incidental), Level 4 (matching), Level 5 (randomized)], follow-up [< 5 years, ≥ 5 years], recidivism definition [Arrest, Charge, Conviction, Multiple definitions, Unspecified], recidivism base rate.
• Treatment characteristics (5 moderators): treatment approach [Behavioral therapy, Cognitive-behavioral therapy, Insight-oriented therapy, Multisystemic therapy, Therapeutic community], treatment setting [Prison, Hospital, Outpatient, Mixed], treatment individualization [Group only, Group mainly, Mixed, Individual mainly, Individual only], treatment specialization [Yes, No], aftercare [Yes, No].
• Individual characteristics (2 moderators): age group [Juveniles, Adult, Mixed, Unclear], risk level [Low-risk, Medium-risk, High-risk, Unclear].

Some other moderators examined by Schmucker and Lösel (2017) were not included in the present analysis because they were poorly documented (treatment mandate, treatment duration, treatment integrity) or unsuitably defined (offender type). None of these moderators were previously reported to be significantly related to sexual recidivism.

### Sensitivity Analyses

Sensitivity analyses were conducted to examine the robustness of the moderator effects when (1) excluding a large sample by Mews et al. (2017), (2) excluding two juvenile samples by Borduin et al. (1990, 2021), (3) excluding all juvenile samples (Borduin et al., 1990; 2021; Guarino-Ghezzi & Kimball, 1998; Lab et al., 1993; Letourneau et al., 2013; Worling et al., 2010), (4) excluding studies with small sample sizes (*n* < 50) (Borduin et al., 1990, 2021; Marshall et al., 1991; Marshall & Barbaree, 1988; Schmid, 1989), and (5) excluding dropouts. Results of the sensitivity analyses are reported in the following sections if they affected the main analysis; otherwise, full details on the sensitivity analyses are provided in the Online Supplementary Materials.

## Results

### Meta-Analysis

**Figure 1.**
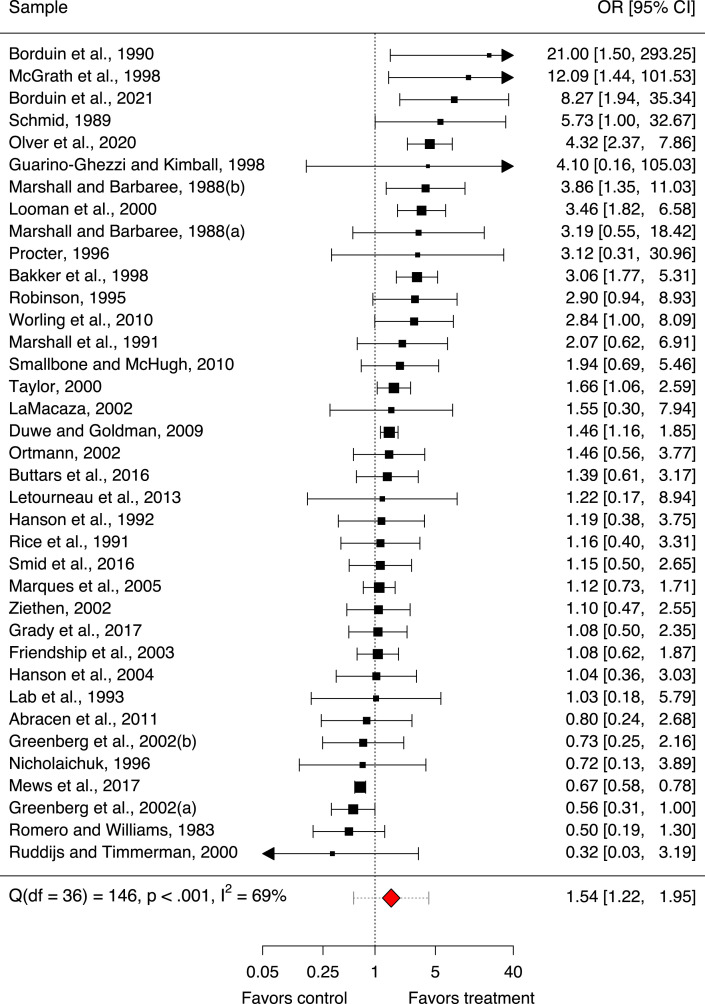
Forest plot sample-specific effects. Forest plot illustrating the 37 sample-specific odds ratios with 95% confidence intervals (OR [95% CI]) included in the updated meta-analysis with respect to sexual recidivism as an indicator of treatment effectiveness in persons with sexual offense histories. Square size is proportionate to the precision of the sample-specific effect sizes. Arrows indicate CIs extending beyond the axis limits. The red diamond represents the mean treatment effect for sexual recidivism with its 95% CIs given in brackets and its 95% prediction interval ([95% PI]) depicted as dotted interval around the diamond.

The main forest plot illustrates the 37 sample-specific ORs [95% CI] of the 35 included studies included in the updated meta-analysis with respect to sexual recidivism as an indicator of treatment effectiveness in persons with sexual offense histories. The size of the squares is proportionate to the precision of the sample-specific effect sizes. The arrows indicate that some CIs extend beyond the axis limits (Figure 1).

The agreement between the sample-specific effect sizes collected in the updated meta-analysis and those collected by Schmucker and Lösel (2017) was compared using the ICC. Since the data collected by Schmucker and Lösel (2017) were unavailable to us, we extracted the ranks of the sample-specific effect sizes (not the sample-specific effect sizes itself, as those were hard to identify) from the forest plot provided in the publication by Schmucker and Lösel (2017, Figure 2). The ICC calculated between the ranks of the updated and the previous sample-specific effect sizes indicated an excellent absolute agreement ( $ICC_{\left(A,1\right)}$ = .971, *p* < .001) considering the guideline for interpreting ICC ( ICC > .90 excellent) (Koo & Li, 2016).

**Figure 2.**
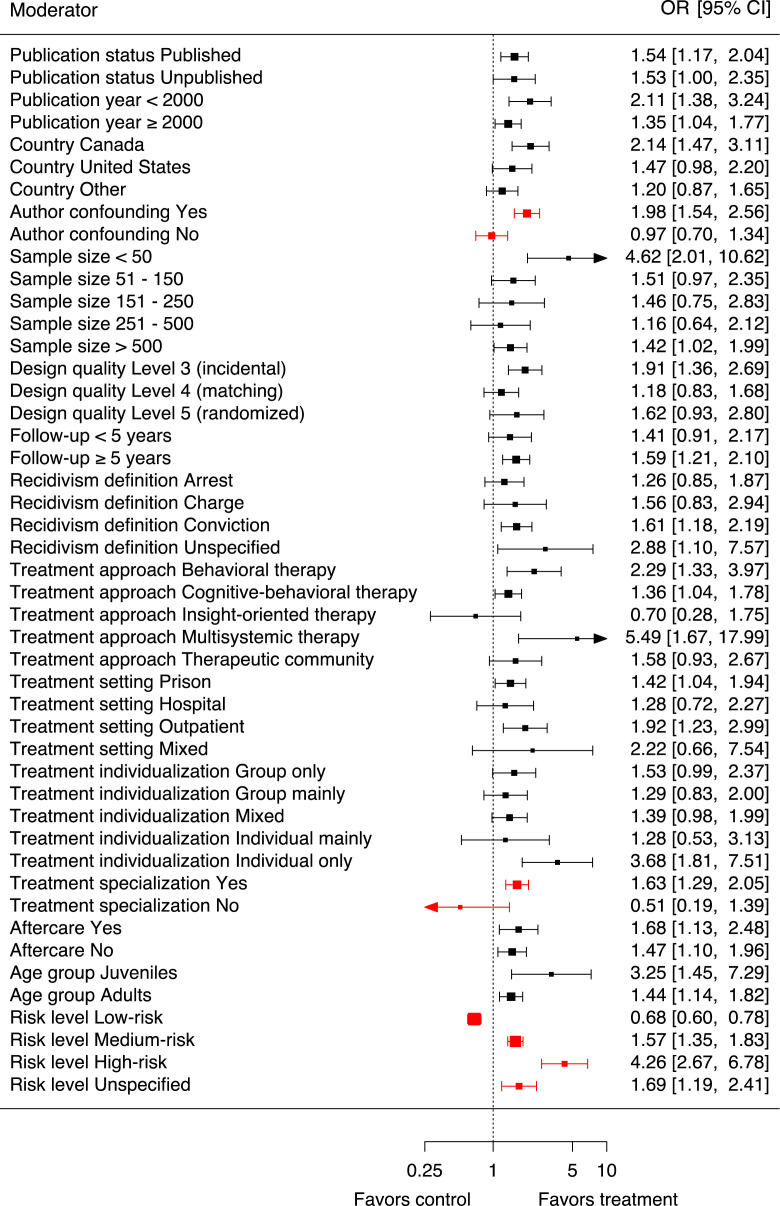
Forest plot moderator-specific effects. Forest plot illustrating the moderator-specific odds ratios (OR [95% CI]) derived from the updated meta-analysis with respect to sexual recidivism as an indicator of treatment effectiveness in persons with sexual offense histories. Square size is proportionate to the precision of the moderator-specific effect sizes. Moderator subgroups between which significant differences were observed and which were robust to all sensitivity analyses are highlighted in red (*p* < .05).

The first forest plot also illustrates the mean treatment effect for sexual recidivism observed in the updated meta-analysis, as represented by the red diamond with the 95% prediction interval ([95% PI]) (Figure 1). The mean treatment effect was significant in terms of an *OR* of 1.54 [95% CI 1.22, 1.95] (*p* < .001). The strength of the effect was small considering Cohen’s equivalent (*d* < .2) (Chen et al., 2010; Cohen, 1988). The 95% PI around the mean treatment effect was wide ([95% PI 0.57, 4.20]) and thus included 1. This indicated that the expected range of true effects in future similar studies will likely fall within this range with a 95% probability and is thus likely to be imprecise.

The mean treatment effect from the 37 samples in the updated meta-analysis (*OR* of 1.54 [95% CI 1.22, 1.95]) was similar to the value that Schmucker and Lösel (2017) reported for their 28 samples (*OR* of 1.41 [95% CI 1.11, 1.78]). A re-analysis of their original 26 studies using our own collected data found an *OR* of 1.54 [95% CI 1.18, 2.01], suggesting slight differences between the two meta-analyses. The mean treatment effect in the eight new studies (*OR* of 1.32 [95% CI 0.82, 2.14]) was not significantly different from our re-analysis of the original 26 studies (*z* = 0.54, *p* = .590).

Sensitivity analyses suggested that the mean treatment effect for sexual recidivism observed in the updated meta-analysis was robust. While excluding the large sample by Mews et al. (2017) (*OR* 1.61 [95% CI 1.28, 2.02], *p* < .001) or excluding dropouts (*OR* 1.56 [95% CI 1.24, 1.98], *p* < .001) slightly strengthened the mean effect, excluding the two small juvenile samples by Borduin et al. (1990, 2021) (*OR* 1.46 [95% CI 1.16, 1.84], *p* = .001), excluding all juvenile samples (*OR* 1.44 [95% CI 1.14, 1.82], *p* = .003), or excluding studies with small sample sizes (*n* < 50) (*OR* 1.41 [95% CI 1.11, 1.78], *p* = .004) slightly weakened the mean effect.

Taken together, these analyses suggest that data collection in the updated meta-analysis was congruent with the data collection by Schmucker and Lösel (2017) and that the current mean treatment effect did not differ substantially from the one that they reported.

### Heterogeneity

Residual heterogeneity across the 35 studies included in the updated meta-analysis was suggested to be substantial (*Q* (*df* = 36) = 146, *p* < .001, $I^{2}$ = 69%). This is larger than that reported by Schmucker and Lösel (2017) ( Q (*df* = 27) = 53, *p* < .01, $I^{2}$ = 48%). Sensitivity analysis suggested that the greater heterogeneity in the updated meta-analysis was partly explained by the large sample by Mews et al. (2017) and excluding that study reduced some of the heterogeneity (*Q* (*df* = 35) = 75, *p* < .001, $I^{2}$ = 56%). The other sensitivity analyses explained less of the heterogeneity, i.e., the two juvenile samples by Borduin et al. (1990, 2021) (*Q* (*df* = 34) = 133, *p* < .001, $I^{2}$ = 68%), all juvenile samples (*Q* (*df* = 30) = 129, *p* < .001, $I^{2}$ = 71%), small samples (*Q* (*df* = 31) = 127, *p* < .001, $I^{2}$ = 70%), and dropouts (*Q* (*df* = 36) = 147, *p* < .001, $I^{2}$ = 69%). Hence, the remaining large heterogeneity of $I^{2}$ = 69% indicated that a substantial percentage of the observed heterogeneity could still not be attributed to sampling error but must be considered as systematic differences between studies. The observed heterogeneity thus corroborated the importance of a moderator analysis that may explain variation in treatment effectiveness in persons with sexual offense histories, as reported in the following sections.

### Moderator Analysis

The following sections provide an overview of the updated moderator analysis. Results are reported first, by detailing the moderator characteristics and second, by specifying their effects on treatment outcome. Table 2 lists the number of samples in each moderator subgroup, the corresponding moderator-specific odds ratios with their 95% CIs. To judge the strength of the moderator effects, i.e., the degree to which predictors may moderate treatment effects, subgroup-contrasts were computed adjusted using the Bonferroni-correction. In Table 2 as well as in the second forest plot (Figure 2) illustrating the moderator-specific ORs, those moderator subgroups between which significant contrasts were observed and which were robust to all sensitivity analyses are highlighted. Notably, Schmucker and Lösel (2017) did not provide information on subgroup-contrasts in their analysis. Details on all subgroup-contrasts and information on the heterogeneity explained by each moderator are provided in the Online Supplementary Materials.

**Table 2.** Updated moderator analysis. Moderator analysis assessing sexual recidivism as indicator of treatment effectiveness. Compared are results reported by Schmucker and Lösel (2017) to the updated moderator analysis. For categorical moderators, effect sizes are reported in terms of odds ratio with 95% confidence intervals (OR [95% CI]). For continuous moderators (cont.), effect sizes were reported in terms of regression weights ( β ) following Schmucker and Lösel (2017). Note that Schmucker and Lösel (2017) collected 28 samples from 26 studies, whereas the updated meta-analysis collected 37 samples from 35 studies. k = number of samples, *n* = sample size. Details on subgroup-contrasts are provided in the Online Supplementary Materials.

	Schmucker and Lösel (2017)			Updated Meta-Analysis		
Publication Characteristics	K	*n*	OR [95% CI]	k	*n*	OR [95% CI]
Publication status						
Published	22	8000		27	10,309	1.54 [95% CI 1.17, 2.04], *p* = .002
Unpublished	7	1781		10	20,085	1.53 [95% CI 1.00, 2.35], *p* = .050
Publication year						
<2000	15	1596		15	3731	2.11 [95% CI 1.38, 3.24], *p* = .001
≥ 2000	14	8185		22	26,663	1.35 [95% CI 1.04, 1.77], *p* = .025
Publication year (cont.)	29	9781		37	30,394	β = 0.04, *z* = 0.27, *p* = .787
Country						
Canada	11	909		13	3855	2.14 [95% CI 1.47, 3.11], *p* < .001
United States	8	3160		11	4382	1.47 [95% CI 0.98, 2.20], *p* = .060
Other	10	5712		13	22,157	1.20 [95% CI 0.87, 1.65], *p* = .260
Author confounding						
Yes	15	6917	1.71 [95% CI 1.18, 2.47]	24	10,972	1.98 [95% CI 1.54, 2.56], *p* < .001
No	11	2864	1.09 [95% CI 0.73, 1.64]	13	19,422	0.97 [95% CI 0.70, 1.34], *p* = .858
Unclear	3					
Descriptive validity (cont.)	28	9765	35	0	30,378	β = 0.55, *z* = 3.82, *p* < .001
Sample characteristics	K	*n*		k	*n*	
Sample size						
<50	7	194	2.14 [95% CI 1.19, 3.84]	5	194	4.62 [95% CI 2.01, 10.62], *p* < .001
51–150	8	1159	1.27 [95% CI 0.75, 2.15]	14	1411	1.51 [95% CI 0.97, 2.35], *p* = .071
151–250	5	791	1.36 [95% CI 0.65, 2.85]	4	801	1.46 [95% CI 0.75, 2.83], *p* = .267
251–500	3	798	1.23 [95% CI 0.59, 2.60]	4	1473	1.16 [95% CI 0.64, 2.12], *p* = .628
≥ 500	6	6839	1.32 [95% CI 0.85, 2.04]	10	26,515	1.42 [95% CI 1.02, 1.99], *p* = .040
Sample size (cont.)	29	9781	β = −-0.05, *z* = −0.29, *p* = .77	37	30,394	β = −-0.11, *z* = −-1.03, *p* = .305
Design quality						
Level 3 (incidental)	15	3493	1.49 [95% CI 1.04, 2.14]	17	4881	1.91 [95% CI 1.36, 2.69], *p* < .001
Level 4 (matching)	8	5282	1.36 [95% CI 0.88, 2.13]	13	22,265	1.18 [95% CI 0.83, 1.68], *p* = .353
Level 5 (randomized)	5	1006	1.36 [95% CI 0.70, 2.62]	7	3248	1.62 [95% CI 0.93, 2.80], *p* = .086
Design quality (cont.)	28	9781	β = −-0.06, *z* = −-0.34, *p* =.73	37	30,394	β = −-0.11, *z* = −-0.79, *p* = .427
Follow-up						
<5 years	16	4535		15	7196	1.41 [95% CI 0.91, 2.17], *p* = .122
≥ 5 years	13	5246		22	23,198	1.59 [95% CI 1.21, 2.10], *p* = .001
Follow-up (cont.)	28	9781	β = −-0.03, *z* = −-0.17	37	30,394	β = 0.20, *z* = 1.40, *p* = .161
Recidivism definition						
Arrest	5	4258	0.98 [95% CI 0.46, 2.09]	12	4784	1.26 [95% CI 0.85, 1.87], *p* = .259
Charge	7	495	1.65 [95% CI 0.93, 2.93]	6	788	1.56 [95% CI 0.83, 2.94], *p* = .165
Conviction	11	4752	1.69 [95% CI 1.12, 2.54]	16	24,546	1.61 [95% CI 1.18, 2.19], *p* = .003
Multiple definitions	3		1.05 [95% CI 0.58, 1.89]			
Unspecified	3	276	1.59 [95% CI 0.63, 4.01]	3	276	2.88 [95% CI 1.10, 7.57], *p* = .032
Recidivism base rate (cont.)	26	9765	β = 0.04, *z* = 2.27, *p* = .02	35	30,378	β = 0.28, *z* = 1.98, *p* = .048
Treatment characteristics	K	*n*		k	n	
Treatment approach						
Behavioral therapy	—	460		6	460	2.29 [95% CI 1.33, 3.97], *p* = .003
Cognitive-behavioral therapy	21	7695	1.38 [95% CI 1.08, 1.75]	21	27,608	1.36 [95% CI 1.04, 1.78], *p* = .025
Insight-oriented therapy	2	338	0.97 [95% CI 0.36, 2.59]	2	338	0.70 [95% CI 0.28, 1.75], *p* = .447
Multisystemic therapy	2	64	21.76 [95% CI 3.70, 128.02]	3	181	5.49 [95% CI 1.67, 17.99], *p* = .005
Therapeutic community	4	1224	1.24 [95% CI 0.69, 2.22]	5	1807	1.58 [95% CI 0.93, 2.67], *p* = .093
Treatment setting						
Prison	10	7791	1.25 [95% CI 0.85, 1.83]	15	25,168	1.42 [95% CI 1.04, 1.94], *p* = .027
Hospital	5	730	1.74 [95% CI 1.04, 2.91]	6	1114	1.28 [95% CI 0.72, 2.27], *p* = .404
Outpatient	12	1260	1.73 [95% CI 1.11, 2.72]	14	1870	1.92 [95% CI 1.23, 2.99], *p* = .004
Mixed	2		0.54 [95% CI 0.19, 1.51]	2	2242	2.22 [95% CI 0.66, 7.54], *p* = .199
Treatment individualization						
Group only	9	3480	1.01 [95% CI 0.66, 1.55]	8	5087	1.53 [95% CI 0.99, 2.37], *p* = .055
Group mainly	8	2155	1.38 [95% CI 0.89, 2.13]	9	17,935	1.29 [95% CI 0.83, 2.00], *p* = .262
Mixed	4	3854	1.87 [95% CI 1.04, 3.36]	12	6380	1.39 [95% CI 0.98, 1.99], *p* = .068
Individual mainly	4	58	1.82 [95% CI 0.87, 3.82]	2	641	1.28 [95% CI 0.53, 3.13], *p* = .585
Individual only	4	234	3.15 [95% CI 1.14, 8.74]	6	351	3.68 [95% CI 1.81, 7.51], *p* < .001
Treatment individualization (cont.)	29	9781	β = 0.41, *z* = 2.47, *p* = .01	37	30,394	β = 0.16, *z* = 1.10, *p* = .271
Treatment specialization						
Yes	26	9377	1.44 [95% CI 1.12, 1.84]	34	29,990	1.63 [95% CI 1.29, 2.05], *p* < .001
No	3	404	1.11 [95% CI 0.45, 2.74]	3	404	0.51 [95% CI 0.19, 1.39], *p* = .189
Aftercare						
Yes	11	3503		12	5007	1.68 [95% CI 1.13, 2.48], *p* = .010
No	18	6278		25	25,387	1.47 [95% CI 1.10, 1.96], *p* = .009
Individual characteristics	K	*n*		k	*n*	
Age group						
Juveniles	5	408	2.97 [95% CI 1.16, 7.59]	6	552	3.25 [95% CI 1.45, 7.29], *p* = .004
Adults	13	9373	1.48 [95% CI 1.03, 2.12]	31	29,842	1.44 [95% CI 1.14, 1.82], *p* = .002
Mixed	1					
Unclear	10					
Age group (cont.)	29	9781	β = −-0.30, *z* = −-1.80, *p* = .07	37	30,394	β = −-0.04, *z* = −-0.26, *p* = .798
Risk level						
Low-risk	8	1700	1.00 [95% CI 0.68, 1.47]	8	17,982	0.68 [95% CI 0.60, 0.78], *p* < .001
Medium-risk	12	6307	1.33 [95% CI 0.96, 1.84]	17	8496	1.57 [95% CI 1.35, 1.83], *p* < .001
High-risk	4	597	3.95 [95% CI 1.77, 8.84]	7	614	4.26 [95% CI 2.67, 6.78], *p* < .001
Unspecified	5	1177		5	3302	1.69 [95% CI 1.19, 2.41], *p* = .004
Risk level (cont.)	29	9781	β = 0.46, *z* = 2.59, *p* = .001	37	30,394	β = 0.33, *z* = 2.62, *p* = .009

### Moderator Characteristics

#### Publication Characteristics

Most studies (73%) were published in scientific journals or books, the remaining were unpublished institutional reports or theses (27%).

Most studies were published after 2000 (59%). The earliest study dates to 1988 (Marshall & Barbaree, 1988), the most recent to 2021 (Borduin et al., 2021). The 10 studies (Abracen et al., 2011; Borduin et al., 2021; Buttars et al., 2016; Grady et al., 2017; Letourneau et al., 2013; Mews et al., 2017; Olver et al., 2020; Smallbone & McHugh, 2010; Smid et al., 2016; Worling et al., 2010) published after 2009 represent the added samples in the updated meta-analysis that had not been included in the meta-analysis by Schmucker and Lösel (2017).

Studies were identified from seven different countries. More than half came from Canada (35%) and the United States (30%). The remaining came from Australia, Germany, Netherland, New Zealand, and the United Kingdom (35%).

Author confounding in terms of authors being involved in the treatment as program directors, supervisors, service providers, or otherwise affiliated with the treatment institution, was observed in 65% of the studies.

Descriptive validity in terms of the accuracy and objectivity of information provided in a publication (Lösel & Köferl, 1989), was heterogeneous across studies. On a 4-point-scale from 0 (very low) to 3 (excellent) the mean was 1.30 (*SD* = 0.66), which is comparable to the mean 1.21 (*SD* = .68) reported by Schmucker and Lösel (2017).

#### Sample Characteristics

Total sample size across studies included 30,394 persons with sexual offense histories (35% treatment groups, 65% control groups). This is nearly three times the sample size included in the meta-analysis by Schmucker and Lösel (2017) (9781 total, 48% treatment groups, 52% control groups). The larger sample size in the updated analysis was mainly due to the inclusion of a very large recent study by Mews et al. (2017), which alone contributed 15,770 (52%) individuals. By contrast, the smallest sample consisted of 16 juveniles evaluated in a study by Borduin et al. (1990). Together with another more recent small juvenile study by Borduin et al. (2021), these two studies contributed only 64 (0.2%) individuals but stood out because of extremely strong effect sizes based on the evaluation of MST. Dropouts contributed 710 (2%) individuals to the treatment groups, as far as information was available.

Study design was reported as incidental assignment justified by statistical procedures to ensure equivalence between treatment and control groups (level 3 on the SMS, 46%), as matching procedure to ensure equivalence between treatment and control groups (level 4, 35%), or as RCT utilizing randomized study designs to assign treatment and control groups (level 5, 19%).

Follow-up period was reported to last ≥ 5 years in most studies (59%). Mean time at risk was 6.7 years (median 5.7 years), ranging from 12 months to 24.8 years.

Recidivism was most commonly defined as re-conviction (43%), followed by (re-)arrest (32%), or new charges (16%). Some studies reported more than one definition to establish whether new offenses had occurred or not; in such cases, the definition that typically comes first in the jurisdiction was evaluated (arrest > charge > conviction); this was done to avoid the small subgroup of multiple definitions as suggested by Schmucker and Lösel (2017) and thus to increase statistical power. The remaining 8% of the studies did not report information on recidivism definition.

Mean (n-weighted) base rate of sexual recidivism was 9.3% in the treatment groups and 13.6% in the control groups. Mean base rates were higher for (re-)arrests (11.9% treatment vs. 14.5% control) and new charges (9.5% treatment vs. 14.1% control) compared to re-convictions (8.9% treatment vs. 13.6% control).

#### Treatment Characteristics

Treatment approaches most commonly evaluated were CBT (57%), followed by earlier behavioral therapeutic approaches (16%), therapeutic communities (14%), MST in juveniles (8%), and insight-oriented approaches (5%).

Treatment took place in institutional settings such as prisons (41%), forensic hospitals (16%), or outpatient settings (38%); some programs reported mixed treatment settings (5%).

Treatment was carried out in about half of the programs using mainly or only group-based formats (46%), some programs utilized both group and individual sessions (32%), and merely 22% focused mainly or only on individual sessions.

The majority of treatment programs were specialized for persons with sexual offense histories (92%). The remaining, though aiming at reducing sexual recidivism, were non-specialized programs.

Aftercare was provided in only 32% of the treatment programs. However, the information provided in the publications was very poor. Some programs mentioned maintenance treatment, maintenance polygraphs, supervised probation or parole, or some form of unspecified aftercare. The remaining 68% of the studies did not provide or did not report aftercare.

#### Individual Characteristics

Most programs treated adults only (84%); in these studies, the mean age was 34.3 years. Programs focusing on juveniles as defined in the publications were less frequently reported (16%); in these studies, the mean age was 14.6 years. In 8% of the studies, information on age was not reported. In all of those studies, however, the sample description allowed for the assumption that adults were addressed; the present analysis therefore counted these samples as adults, in contrast to Schmucker and Lösel (2017) who considered these samples as separate category (‘unclear’ age). One study (Ruddijs & Timmerman, 2000) included both adults and a small percentage of juveniles (9%) with an overall mean age of 34 years; the present analysis therefore counted this sample as adults, in contrast to Schmucker and Lösel (2017) who considered this sample as separate category (‘mixed’ age). This age subgrouping was done to avoid very small subgroups and thus to increase statistical power.

Risk level was rated as low-risk (22%), medium-risk (46%), or high-risk (19%). If information on risk level was reported in the primary studies based on individual risk assessments, such as the Static-99 (Harris et al., 2003), the Static-99R (Phenix et al., 2016), the Risk Matrix score (Ross & Loss, 1991), or the BARS (Brief Actuarial Risk Scale) (Olver et al., 2013), it was used in the updated meta-analysis, which was possible in 12 (34%) of the cases. Following Schmucker and Lösel (2017), in cases, where there was no proper risk assessment reported in the studies, the Rapid Risk Assessment for Sex Offence Recidivism (RRASOR) (Hanson, 1997) was used to evaluate mean risk level based on information collected from the publications; this was possible in 18 (51%) of the cases. Mean risk level derived using the RRASOR, however, represents only a rough estimate and cannot be compared to risk assessment done in individuals. The RRASOR is further only recommended for persons with sexual offense histories from the age of 18 years upwards. This should be considered when interpreting mean risk level in the juvenile samples, all of which, except one (Lab et al., 1993), were rated using the RRASOR in the updated meta-analysis. Another five (14%) studies did not allow for any risk estimate.

### Moderator Effects

The following sections report the effects of the above-described moderators on sexual recidivism as an indicator of treatment effectiveness. In the text, we only report statistically significant effects ( p < .05); details on all moderator effects and the corresponding subgroup-contrasts are provided in Table 2 and the Online Supplementary Materials, respectively.

#### Publication Characteristics

Publication status was associated with similar significant treatment effects for published ( OR 1.54 [95% CI 1.17, 2.04], *p* = .002, k = 27, n = 10,309) and unpublished ( OR 1.53 [95% CI 1.00, 2.35], *p* = .050, k = 10, n = 20,085) studies, though the latter was based on fewer studies and was not statistically significant; the subgroup-contrast was not significant. Schmucker and Lösel (2017) did not report effect sizes corresponding to publication status but mentioned that the moderator had overall no effect ( Q (*df* = 1) = .01, *p* = .94).

Publication year was not linearly related to treatment effects. Both, studies published before 2000 ( OR 2.11 [95% CI 1.38, 3.24], *p* < .001, k = 15, n = 3731) and after 2000 ( OR 1.35 [95% CI 1.04, 1.77], *p* = .025, k = 22, n = 26,663) were associated with significant treatment effects, indicating no advantage in earlier or more recent decades. Schmucker and Lösel (2017) did not report the corresponding effect sizes.

Country of origin was associated with a significant treatment effect for Canada ( OR 2.14 [95% CI 1.47, 3.11], *p* < .001, k = 13, n = 3855), which was robust to all sensitivity analyses. There was also a marginally significant treatment effect suggested for the Unites States ( OR 1.47 [95% CI 0.98, 2.20], *p* = .060, k = 11, n = 4382), which became non-significant after excluding the two small juvenile samples on MST by Borduin et al. (1990, 2021). None of the subgroup-contrast were statistically significant. Schmucker and Lösel (2017) did not report the corresponding effect sizes.

The strongest moderating effect among the publication characteristics was observed for author confounding. In line with Schmucker and Lösel (2017), this indicated that authors involved in or affiliated with the treatment programs reported significantly larger treatment effects ( OR 1.98 [95% CI 1.54, 2.56], *p* < .001, k = 24, n = 10,972) compared to outcomes reported by independent authors, with the difference being significant (*z* = 3.40, *p* < .001) and robust to all sensitivity analyses. Author confounding reduced a great amount of residual heterogeneity by 48%, as measured by the reduction of residual heterogeneity compared to the main effect (Online Supplementary Materials).

Another strong moderating effect resulted for descriptive validity ( β = 0.55, *z* = 3.82, *p* < .001, k = 35, n = 30,378), which was also robust to all sensitivity analyses. In line with Schmucker and Lösel (2017), this suggested that unsatisfactory quality in research reporting was linearly associated with worse outcomes.

#### Sample Characteristics

Sample size was not linearly related to treatment effects. In line with Schmucker and Lösel (2017), there was however a significant small-study effect ( n < 50) ( OR 4.62 [95% CI 2.01, 10.62], *p* < .001, k = 5, n = 194), suggesting greater treatment effects in smaller compared to larger samples he subgroup difference was marginally significant (*z* = −2.57, *p* = .071), but became non-significant after excluding the two small juvenile samples on MST by Borduin et al. (1990, 2021). This marginally small-study effect was supported by a non-significant Egger’s test suggesting funnel plot asymmetry (*z* = 1.85, *p* = .064). A funnel plot is provided in the Online Supplementary Materials.

Design quality was also not significantly linearly related to treatment effects. In accordance with Schmucker and Lösel (2017), there was a significant treatment effect for level 3 designs on the SMS ( OR 1.91 [95% CI 1.36, 2.69], *p* < .001, k = 17, n = 4881), suggesting a negative tendency of larger treatment effectiveness reported in incidental study designs (level 3) compared to matched trials (level 4) or RCTs (level 5); though subgroup-contrasts indicated no significant difference between levels. As noted by Schmucker and Lösel (2017), this result both reflects the low number and high heterogeneity among RCTs with the two small juvenile samples by Borduin et al. (1990, 2021) showing extremely strong treatment effects, whereas the remaining five RCTs revealed weaker or even negative effects (Letourneau et al., 2013; Marques et al., 2005; Ortmann, 2002; Robinson, 1995; Romero & Williams, 1983). Consequently, sensitivity analysis showed that excluding the two studies by Borduin et al. (1990, 2021) enhanced the negative linear association between decreasing design quality and increasing treatment effectiveness, though never reaching significance level.

Follow-up length was also not significantly linearly related to sexual recidivism. Though the treatment effect for longer follow-up periods ≥ 5 years was significant ( OR 1.59 [95% CI 1.21, 2.10], *p* < .001, k = 22, n = 23,198) and slightly larger as opposed to shorter follow-up periods <5 years ( OR 1.41 [95% CI 0.91, 2.17], *p* = .122, k = 15, n = 7196), the latter was also marginally significant. This indicated that there was essentially no significant difference depending on follow-up duration, which was robust to all sensitivity analyses.

Recidivism definition was associated with a significant effect for re-conviction ( OR 1.61 [95% CI 1.18, 2.19], *p* = .003, k = 16, n = 24,546), whereas no such effects were observed for (re-)arrest or new charges. In line with Schmucker and Lösel (2017), however, none of the subgroup differences were statistically significant and sensitivity analyses did not suggest otherwise.

Mean base rate of sexual recidivism was marginally linearly related to treatment effects ( β = 0.28, *z* = 1.98, *p* = .048, k = 35, n = 30,378), in line with Schmucker and Lösel (2017). The effect was, however, not robust to any of the sensitivity analyses.

#### Treatment Characteristics

Treatment programs based on CBT ( OR 1.36 [95% CI 1.04, 1.78], *p* = .025, k = 21, n = 27,608), MST ( OR 5.49 [95% CI 1.67, 17.99], *p* = .005, k = 3, n = 181), and earlier behavioral approaches ( OR 2.29 [95% CI 1.33, 3.97], *p* = .003, k = 6, n = 460) were suggested to have significant treatment effects, whereas insight-oriented approaches or therapeutic communities did not. Sensitivity analysis indicated that the effect of MST was a function of the two small juvenile samples on MST by Borduin et al. (1990, 2021); after excluding the two studies there was only one study left making a comparison infeasible. There was no indication of a clear advantage of one treatment approach over others, as none of the subgroup-contrasts were statistically significant.

Treatment setting was associated with significant effects for treatments carried out in prisons ( OR 1.42 [95% CI 1.04, 1.94], *p* = .027, k = 15, n = 25,168) and outpatient settings ( OR 1.92 [95% CI 1.23, 2.99], *p* = .004, k = 14, n = 1870); the contrast to other settings was however not significant. Sensitivity analysis suggested the effect of outpatient settings become non-significant after excluding juvenile samples or studies with small sample sizes. While Schmucker and Lösel (2017) reported a significant effect for treatments provided in forensic hospitals, the present analysis found no indication for such an effect.

Treatment individualization was related to better outcomes, though the updated analysis did not observe the significant linear relation reported by Schmucker and Lösel (2017). Programs that had a strong individualized approach ( OR 3.68 [95% CI 1.81, 7.51], *p* < .001, k = 6, n = 351) appeared to perform better than programs carried out in group-based or mixed formats, though differences between formats were non-significant. The effect of treatment individualization was stable across sensitivity analyses. The observation of better outcomes with increasing treatment individualization is also supported by the findings on SOTP (Dennis et al., 2012; Völlm, 2018).

Treatment specialization was the strongest predictor among the treatment characteristics. Programs that provided specialized treatment for persons with sexual offense histories ( OR 1.63 [95% CI 1.29, 2.05], *p* < .001, k = 34, n = 29,990) were suggested to result in larger treatment effects compared to non-specialized programs. Indeed, after non-specialized treatment a non-significant increase in sexual recidivism was observed ( OR 0.51 [95% CI 0.19, 1.39], *p* = .189, k = 3, n = 404), suggesting that non-specialized programs are ineffective or even do more harm than good. The strength of the specialization effect was represented by a significant subgroup-contrast (*z* = 2.21, *p* = .027) and robustness to all sensitivity analyses. Surprisingly, this moderator did not reduce much of the residual heterogeneity, i.e., only 2%. Schmucker and Lösel (2017) reported no effect of treatment specialization.

Aftercare was not associated with a significant subgroup difference. Both, programs providing some form of aftercare ( OR 1.68 [95% CI 1.13, 2.48], *p* = .010, k = 12, n = 5007) and those not providing aftercare ( OR 1.47 [95% CI 1.10, 1.96], *p* = .009, k = 25, n = 25,387) revealed significant treatment effects. Schmucker and Lösel (2017) did not report the corresponding effect sizes.

#### Individual Characteristics

Age was not linearly related to treatment effects. Both, juveniles ( OR 3.25 [95% CI 1.45, 7.29], *p* = .004, k = 6, n = 552) and adults ( OR 1.44 [95% CI 1.14, 1.82], *p* = .002, k = 31, n = 29,842) were suggested to benefit from the treatment, with the subgroup difference being not significant.

Risk level was suggested the strongest predictor for sexual recidivism as an indicator of treatment effectiveness. There was a strong linear effect on sexual recidivism ( β = 0.33, *z* = 2.62, *p* = .009, k = 37, n = 30,394), indicating greater benefit from treatment in higher compared to lower risk levels. Indeed, while high-risk individuals ( OR 4.26 [95% CI 2.67, 6.78], *p* < .001, k = 7, n = 614) and medium-risk individuals ( OR 1.57 [95% CI 1.35, 1.83], *p* < .001, k = 17, n = 8496) demonstrated lower sexual recidivism after treatment, low-risk individuals ( OR 0.68 [95% CI 0.60, 0.78], *p* < .001, k = 8, n = 17,982) even demonstrated an increase in sexual recidivism as expressed by an OR below one after treatment. All subgroup-contrasts were significant, that is, between high- and medium-risk individuals (*z* = −3.98, *p* < .001), between high- and low-risk individuals (*z* = −7.40, *p* < .001), and between medium- and low-risk individuals (*z* = 8.00, *p* < .001), thus supporting the strong linear effect of pre-treatment risk level. This moderator reduced the greatest amount of residual heterogeneity among all predictors, i.e., 73%, compared to the mean effect (Online Supplementary Materials). Sensitivity analyses suggested that the effect was robust to all sensitivity analyses.

A summary table lists the three most robust predictors suggested by the updated meta-analysis (Table 3).

**Table 3.** Summary updated moderator meta-analysis. Listed are the three predictors that were suggested to moderate treatment effectiveness in the updated meta-analysis based on significant subgroup-contrasts derived from general linear hypothesis (GLH) testing. A summary statement is provided regarding the direction in which the corresponding moderator affects treatment effectiveness. These three predictors may be viewed as the most robust factors moderating treatment effectiveness in persons with sexual offense histories found in the updated meta-analysis.

Moderator	Direction
Author confounding	Larger treatment effects in studies with author involvement in the treatment process
Treatment specialization	Larger treatment effects in specialized compared to non-specialized treatment programs
Risk level	Larger treatment effects in higher-risk compared to low-risk samples

## Discussion

The present meta-analysis provides an update of the recent meta-analysis by Schmucker and Lösel (2017). The sample size evaluated in the updated meta-analysis increased from 9781 to 30,394 cases compared to the previous analysis by Schmucker and Lösel (2017). It has to be emphasized, though, that mainly one large sample was responsible for that increase in sample size (Mews et al., 2017, 15,770 cases). In accordance with Schmucker and Lösel (2017), the mean effect for sexual recidivism as an indicator of treatment effectiveness was small with an OR of 1.54 [95% CI 1.22, 1.95] (*p* < .001). This equated to a reduction in sexual recidivism after treatment of 31.8% (mean n-weighted sexual recidivism rate of 9.3% in treatment vs. 13.6% in control groups). The reduction reported by Schmucker and Lösel (2017) was slightly lower in terms of 26.4% (mean n-weighted sexual recidivism rate of 10.1% in treatment vs. 13.7% in control groups) corresponding to an OR of 1.41 [95% CI 1.11, 1.78] (*p* = .005).

The substantial residual heterogeneity (*Q* (*df* = 36) = 146, *p* < .001) observed with an $I^{2}$ index of 69% called for a thorough moderator analysis to evaluate the factors that might reduce heterogeneity depending on the various conditions. Results of the moderator analysis suggested three predictors of interest, i.e., risk level, treatment specialization, and author confounding. All three moderators revealed significant subgroup-contrasts indicating a plausible moderation of the mean treatment effect. That is, higher compared to lower risk, specialized compared to non-specialized treatment, and the presence of author confounding compared to no author confounding, were suggested to increase the mean treatment effect. Further, all three moderators were robust to sensitivity analyses. And together the three moderators explained a great amount of heterogeneity, with author confounding (48%) and risk level (73%) explaining more than treatment specialization (2%). These three predictors may thus be viewed as the most robust factors moderating treatment effectiveness in persons with sexual offense histories found in the updated meta-analysis.

Together, the present results are overall in line with the findings reported by Schmucker and Lösel (2017), though some of the observations were more pronounced, such as the effect of risk level, and some only became significant in the present analysis, such as the effect of treatment specialization. The updated meta-analysis thus sharpened the evidence on the importance of these moderators in the context of sexual recidivism as an indicator of treatment effectiveness in persons with sexual offense histories.

The observations made in the updated meta-analysis corroborated the relevance of the RNR model (Bonta & Andrews, 2007). The importance of risk level and treatment specialization correspond to the first and second principle of the RNR model. While the first principle, the risk principle, states that treatment allocation should be guided by an individuals’ risk level, the second principle, the need principle, states that treatment should be allocated to the individuals’ criminogenic needs. The third RNR principle, the responsivity principle, which states that treatment should be delivered attuned to the learning and motivational style of offenders, and which has previously best been proven for CBT (Hanson et al., 2009a, 2009b), was not supported by the present analysis as there was no indication of a clear advantage of one treatment approach over others.

The first principle of the RNR model states that higher-compared to lower-risk individuals are more likely to benefit from treatment. Following the principle, intensive treatment may therefore be reserved for higher-risk individuals, while it may be inefficient or even increase recidivism in low-risk individuals (Lovins et al., 2009; Wilson et al., 2007). This observation was corroborated by the updated meta-analysis pointing to a strong negative effect for treatment involving low-risk offenders, which may result in an increase in sexual recidivism. This effect became more pronounced in the updated compared to the previous meta-analysis by Schmucker and Lösel (2017).

The updated meta-analysis pointing to a strong negative effect for treatment involving low-risk offenders, which may result in an increase in sexual recidivism.

The observations that higher compared to lower pre-treatment risk levels are more likely to result in greater treatment effectiveness, and hence reduced sexual recidivism, are however not generally supported. Some meta-analyses shared that observation (Landenberger & Lipsey, 2005; Schmucker & Lösel, 2015, 2017), whereas others did not (Hanson et al., 2009a; 2009b; Ter Beek et al., 2018). Interpreting the role of risk level should therefore be made with caution. For example, it has been argued that risk level may be biased by the risk scale measured, such that more homogeneous categories differentiating low-, medium-, versus high-risk individuals (Schmucker & Lösel, 2017) may have greater statistical power compared to dichotomous categories separating only low-versus high-risk individuals (Hanson et al., 2009a, 2009b). The source of risk rating may also play a role, such that ratings based on individual risk assessments (e.g., Static-99) should generally be preferred over those based on aggregated risk assessments (e.g., RRASOR), as done in the present work. Since the updated analysis judged risk level in 51% of the samples based on aggregated risk assessments and only 34% based on individual risk assessments, with another 14% not allowing for any risk estimate, the presented risk ratings should be considered only a rough estimate of mean risk level. Furthermore, it should be mentioned that the cut-offs into low, medium, or high are dependent on the respective tool and may have been entirely developed based on risk distributions. As such, the present rating is not an objective or consistent measure of risk, since the source of risk ratings differed between primary studies. To over come this issue, a framework for standardizing risk communication independent of any particular offender risk scale has been suggested more recently (Hanson et al., 2017a, 2017b).

Further, methodological biases in treatment evaluation may be discussed. For example, it has been argued that the relationship between risk level and treatment effectiveness may not be linear, making causal inferences about treatment effectiveness difficult. High-psychopathic individuals, who also qualify as high-risk on risk tools for sexual reoffending have been reported to be particularly difficult to treat (Lösel, 1998), because they show significantly higher rates of treatment non-completion (30%) than low-psychopathy men (6%), and may therefore often be excluded from treatment programs, although they do show evidence of therapeutic benefit (Sewall & Olver, 2019). On the other side, recidivism rates for individuals qualifying as low-risk are typically so small that treatment may not add much to further reduce sexual recidivism (Schmucker & Lösel, 2017). This may induce prevalence-related biases in the evaluation (Austin et al., 2002). Also, it has been suggested that because of limited resources treatment may be offered preferably to those who are more likely to be amenable to treatment, e.g., those admitting responsibility for sexual offenses, and thus less likely to re-offend in the first place (Mailloux et al., 2003; Reitzel & Carbonell, 2006). This may induce treatment-benefit biases. Finally, the relation between risk level and treatment effectiveness may also be considered from an economic perspective. Because high-compared to low-risk individuals may be expected to require more treatment, the cost per treatment to the criminal justice system may, on an expected value basis, be higher for high-than low-risk populations (Aos et al., 2006; Bourgon & Armstrong, 2005). This may induce cost-benefit biases in the evaluation.

The second principle of the RNR model states that to effectively reduce recidivism, treatment programs should target criminogenic needs, which are dynamic risk factors related to subsequent offending, such as substance use or an antisocial lifestyle (Andrews et al., 1990). The significant effect of treatment specialization observed in the updated meta-analysis extends the meta-analysis by Schmucker and Lösel (2017), who reported no effect of treatment specialization. Previous studies on the need principle suggested that programs successfully addressing criminogenic needs were associated with an average 19% decrease in sexual recidivism, while treatments focusing on non-criminogenic needs were found to slightly increase recidivism by about 1% (Andrews & Bonta, 2006). These observations were corroborated in the updated meta-analysis, where specialized programs were suggested to significantly decrease sexual recidivism by 34%, but non-specialized treatments non-significantly increased sexual recidivism by 86% after treatment. Targeting interventions to criminogenic needs therefore remains an important aspect in the treatment of persons with sexual offense histories.

Author confounding was also observed in the present analysis. Authors being involved in the treatment such as program directors, supervisors, service providers, or otherwise affiliated with the treatment institution, reported larger mean treatment effects compared to studies reported by independent authors. Author involvement is a potentially serious confounder for the outcome of a study and can lead to publication and reporting biases (Abou-Setta et al., 2019). Though deficits in descriptive validity were not observed in the updated meta-analysis. Author confounding is, however, not specifically related to programs providing treatment to persons with sexual offense histories but is a frequently reported problem in scientific research (Dunn et al., 2016). A more specific effect of author involvement only considering author directly involved in treatment, such as psychologists or supervisors, was not conducted as only few studies (Borduin et al., 1990, 2021) declared this level of treatment-related author involvement.

Design quality, though not emerging as an important predictor in the updated meta-analysis, requires some discussion, as it was the main methodological advantage of the work by Schmucker and Lösel (2017) compared to other meta-analyses. To comply with the coding scheme reported by Schmucker and Lösel (2017), we used the SMS scale (Farrington et al., 2002), which rates design quality of criminological interventions in general. The rating guide provided by the Collaborative Outcome Data Committee’s Guidelines for the Evaluation of Sexual Offender Treatment Outcome Studies (CODC Guidelines) (Beech et al., 2007a), may also been suited as it was specifically developed for evaluating studies in persons with sexual offenses. In line with Schmucker and Lösel (2017), the updated meta-analysis suggested a tendency of a negative relation between design quality and treatment effects, indicating larger effectiveness in studies applying level 3 compared to level 4 or level 5 designs on the SMS. Together, this suggested that randomized trials yielded lower treatment effectiveness compared to designs with weaker quality. This point also relates to the observation of greater effectiveness in small and author initiated studies. Schmucker and Lösel (2017) pointed out that the findings may be biased by the relatively large proportion of the qualitatively weaker level 3 studies. This potential bias became expectedly stronger after excluding more of the RCTs in the sensitivity analyses. Similar observations have been made earlier and raised a debate on the evaluation of RCTs. On the one hand, such findings raised doubts on the appropriateness of RCTs in this field (Lösel & Schmucker, 2005; Marshall & Marshall, 2007; Schmucker & Lösel, 2017; Seto et al., 2008). For example, RCTs have been suggested to be difficult or even impossible to carry out in the treatment of individuals with a history of sexual offending because various countries require mandatory treatment depending on the seriousness of the sexual offense, which makes the formation of randomized control groups impossible for legal or practical reasons. Furthermore, the relatively low base rate of sexual reoffending as registered in official records may require large samples to reveal significant effects, which may be costly for RCTs. Moreover, RCTs may not adequately address the practice of psychotherapy (Hollin, 2008; Seligman & Levant, 1998). Finally, threats to internal validity to guarantee full equivalence of treatment and control groups may also occur in RCTs (Lösel, 2007; Marques et al., 2005). On the other hand, RCTs are still recommended as the gold-standard (Jones & Podolsky, 2015), and the difficulties in generating reliable RCTs in the field should not be confused with low reliability of the results (Beaudry et al., 2021; Dennis et al., 2012; Schmucker & Lösel, 2017; Völlm, 2018). As noted earlier by the CODC (Beech et al., 2007b), knowledge is cumulative and both RCTs and lower quality studies are needed to form convincing evidence. Together, this calls for the necessity of conducting more RCTs to validate the rather low effectiveness of treatment in persons with sexual offense histories.

Though follow-up length was not found to be associated with the treatment effect in the present analysis, this moderator is often a topic of discussion. The sometimes observed phenomenon of increasing recidivism with increasing follow-up length has been explained by the fact that individuals are tracked for lengthier periods of time in which recidivism can occur; following this argumentation, longer follow-up periods may provide more accurate estimates of recidivism or otherwise desistance from crime (Fazel & Wolf, 2015). On the other hand, longer follow-up periods may also allow time for other influences to evolve that may have positive impacts on the life of an offender, thus supposedly reducing recidivism (Schmucker & Lösel, 2017). Thus, increasing follow-up periods and different life situations may make it difficult to determine whether recidivism indeed reflects effects of the treatment provided if proper knowledge on length of aftercare is missing or unconsidered.

The present analysis has several methodological limitations. First, the data of the updated meta-analysis were collected by one of the authors (L.H.). We therefore were not able to provide a measure of inter-rater reliability of the updated data. However, though it was not possible to estimate the true agreement between the data collection in the updated meta-analysis and that by Schmucker and Lösel (2017), because the latter was not available to the current authors, we were able to estimate the ICC between the ranks of the sample-specific effects sizes ( $ICC_{\left(A,1\right)}$ = .971, *p* < .001). Data collection may therefore be considered reliable, still, slight differences in sample-specific effects sizes may have contributed to slight differences in the mean treatment effects in our re-analysis. Second, the moderator variables collected from the primary studies contained missing values due to insufficient information available. When interpreting the present findings, it should therefore be kept in mind that missing values may have biased the results. Third, the subgroup-contrasts examined in the moderator analysis were Bonferroni-corrected to counteract the problem of multiple comparisons. Though Bonferroni correction is the simplest method for counteracting this, it is a conservative method that gives greater risk of failure to reject a false null hypothesis than other methods as it ignores potentially valuable information, such as the distribution of *p*-values across all contrasts. It should therefore be considered that the application of alternative methods, such as the Holm–Bonferroni method, the Šidák correction, or the false discovery rate (FDR) (Glickman et al., 2014; Holm, 1979), might have led to slightly different conclusions. Last, the present moderator analysis evaluated only the main moderator effects. Moderator analysis may, however, also be applied for the evaluation of interaction effects between moderators. For example, risk level of the sample may be related to the settings in which treatment takes place. Examining interactions, however, make the interpretation of the resulting terms complex, depending on how many predictors are aimed to be included in one model. It also requires large number of samples. Therefore, the present analysis refrained from assessing interaction terms to keep the findings comprehensible and applicable in forensic practice.

Taken together, the updated meta-analysis suggested that persons with sexual offense histories who receive treatment are less likely to reoffend than those not receiving treatment. Though the treatment effectiveness was suggested to be still small, not all treatments were suggested to be equally effective and higher risk individuals were suggested to benefit most. The updated meta-analysis may thus provide support for practitioners and decision-makers in gauging the current evidence on treatment effectiveness in persons with sexual offense histories as measured by offense recidivism. More specifically, the current finding may help researchers to implement and carry out informative, methodologically sound evaluations of ongoing treatment programs. Ideally, such future studies should also include more proximal indicators of treatment success, such as key ingredients of different treatment approaches and the role of other individual characteristics within high-quality differential studies to further knowledge development about “what works best for whom?” (Tyler et al., 2021).

## Supplemental Material

Supplemental Material - Moderators of Sexual Recidivism as Indicator of Treatment Effectiveness in Persons With Sexual Offense Histories: An Updated Meta-analysis Supplemental Material for Moderators of Sexual Recidivism as Indicator of Treatment Effectiveness in Persons With Sexual Offense Histories: An Updated Meta-analysis by Lisa Holper, Andreas Mokros and Elmar Habermeyer in Sexual Abuse
