# Supplementary material for: Moderators of Sexual Recidivism as Indicator of Treatment Effectiveness in Persons With Sexual Offense Histories: An Updated Meta-analysis
Source: Sex Abuse. 2023 Mar 16;36(3):255–91. doi: 10.1177/10790632231159071 (PMC10880427; doi:10.1177/10790632231159071)
Supplement: Supplemental Material - Moderators of Sexual Recidivism as Indicator of Treatment Effectiveness in Persons With Sexual Offense Histories: An Updated Meta-analysis [file sj-pdf-1-sax-10.1177_10790632231159071.pdf]

Supplementary materials

Moderators of sexual recidivism as indicator of treatment effectiveness in persons with sexual offense histories: an updated meta-analysis

Holper, Lisa [1,*], Mokros, Andreas [2], Habermeyer, Elmar [1]

[1] Department of Forensic Psychiatry, University Hospital of Psychiatry Zurich, Zurich, Switzerland

[2] FernUniversität in Hagen, Faculty of Psychology, Chair of Personality, Legal Psychology, and Assessment, Hagen, Germany

Table of Contents

PRISMA **2**

ICC **4**

Meta-analysis **5**

Moderator coding scheme **8**

  Publication characteristics 8

  Sample characteristics 8

  Treatment characteristics 9

  Individual characteristics 9

Moderator sensitivity analysis **11**

References **17**

PRISMA

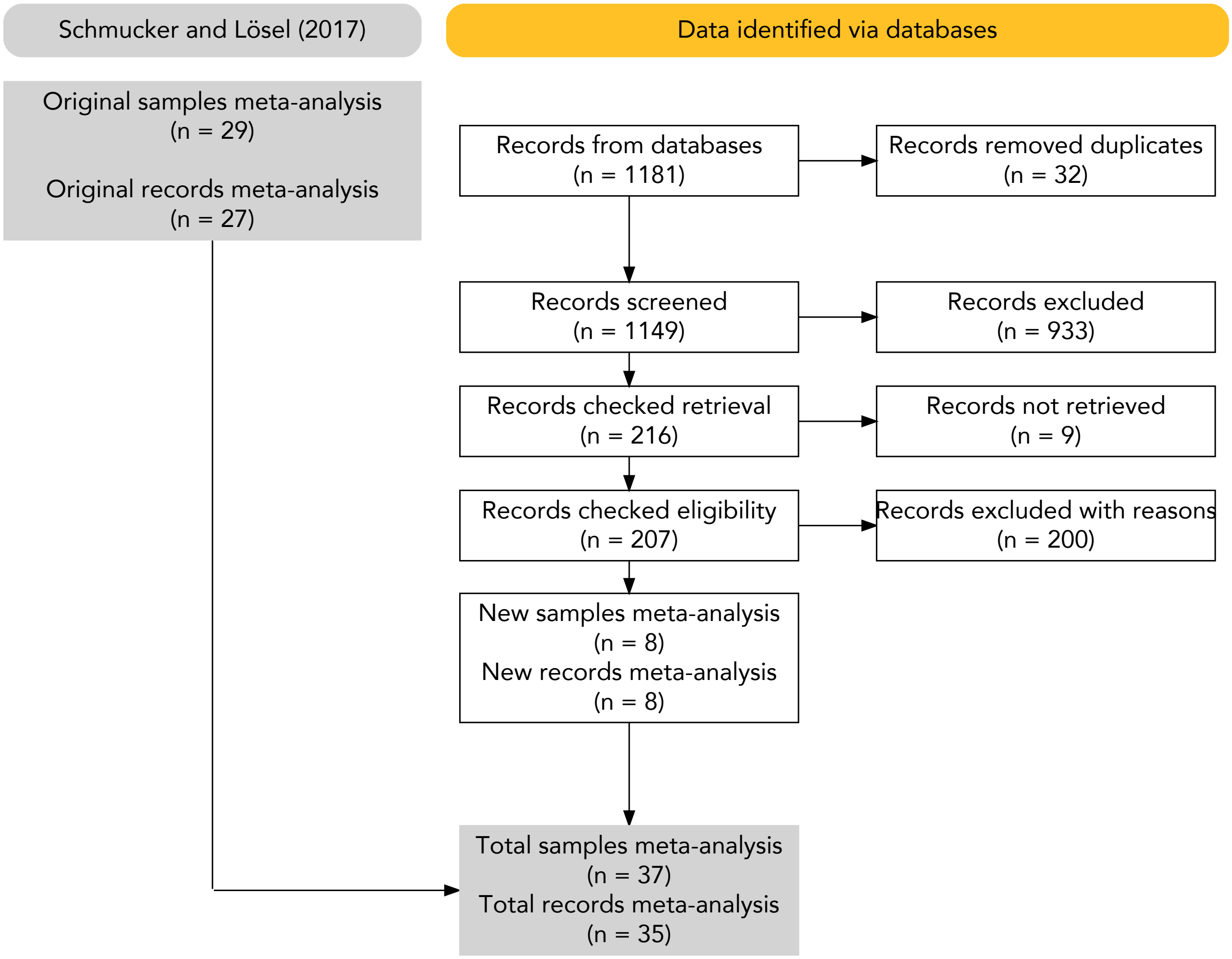

Figure 1: PRISMA flow diagram. *PRISMA flow diagram illustrating the study selection process. Generated using the `PRISMA2020` package (Haddaway et al., 2021) in R programming language (Team, 2022).*

PRISMA flow statement detailing the study selection process.

- **Original studies: N = 27** studies (Bakker et al., 1998; Borduin et al., 1990, 2009; Duwe & Goldman, 2009; Friendship et al., 2003; Greenberg et al., 2002; Guarino-Ghezzi & Kimball, 1998; Hanson et al., 1992; Hanson et al., 2004; Lab et al., 1993; LaMacaza, 2002; Looman et al., 2000; Marques et al., 2005; Marshall et al., 1991; Marshall & Barbaree, 1988; McGrath et al., 1998; Nicholaichuk, 1996; Ortmann, 2002; Procter, 1996; Rice et al., 1991; Romero & Williams, 1983; Ruddijs & Timmerman, 2000; Schmid, 1989; Taylor, 2000; Worling & Curwen, 2000; Ziethen, 2002) identified and judged being eligible by Schmucker and Lösel (2017). Two of these original studies were updated with more recent publications on the same samples, one (Worling et al., 2010) as recommended by Schmucker and Lösel (2017), the other (Borduin et al., 2021) identified by the current authors.

- **Recommended studies: N = 6** studies (Abracen et al., 2011; Grady et al., 2017; Letourneau et al., 2013; Olver et al., 2020; Smallbone & McHugh, 2010; Smid et al., 2016) recommended by Schmucker and Lösel (2017) as being eligible for updating the meta-analysis. Two of these recommended studies were updated with more recent publications on the same samples (Grady et al., 2017; Olver et al., 2020) identified by the current authors.

- **Additional studies: N = 2** studies (Buttars et al., 2016; Mews et al., 2017), which were published after the completion of meta-analysis by Schmucker and Lösel (2017), were identified and found to be eligible based on database search and other recent meta-analyses (Gannon et al., 2019; Lösel, 2020).

- **Included studies: N = 35** total studies (Abracen et al., 2011; Bakker et al., 1998; Borduin et al., 1990, 2021; Buttars et al., 2016; Duwe & Goldman, 2009; Friendship et al., 2003; Grady et al., 2017; Greenberg et al., 2002; Guarino-Ghezzi & Kimball, 1998; Hanson et al., 1992; Hanson et al., 2004; Lab et al., 1993; LaMacaza, 2002; Letourneau et al., 2013; Looman et al., 2000; Marques et al., 2005; Marshall et al., 1991; Marshall & Barbaree, 1988; McGrath et al., 1998; Mews et al., 2017; Nicholaichuk, 1996; Olver et al., 2020; Ortmann, 2002; Procter, 1996; Rice et al., 1991; Robinson, 1995; Romero & Williams, 1983; Ruddijs & Timmerman, 2000; Schmid, 1989; Smallbone & McHugh, 2010; Smid et al., 2016; Taylor, 2000; Worling et al., 2010; Ziethen, 2002) were included in the updated meta-analysis.

ICC

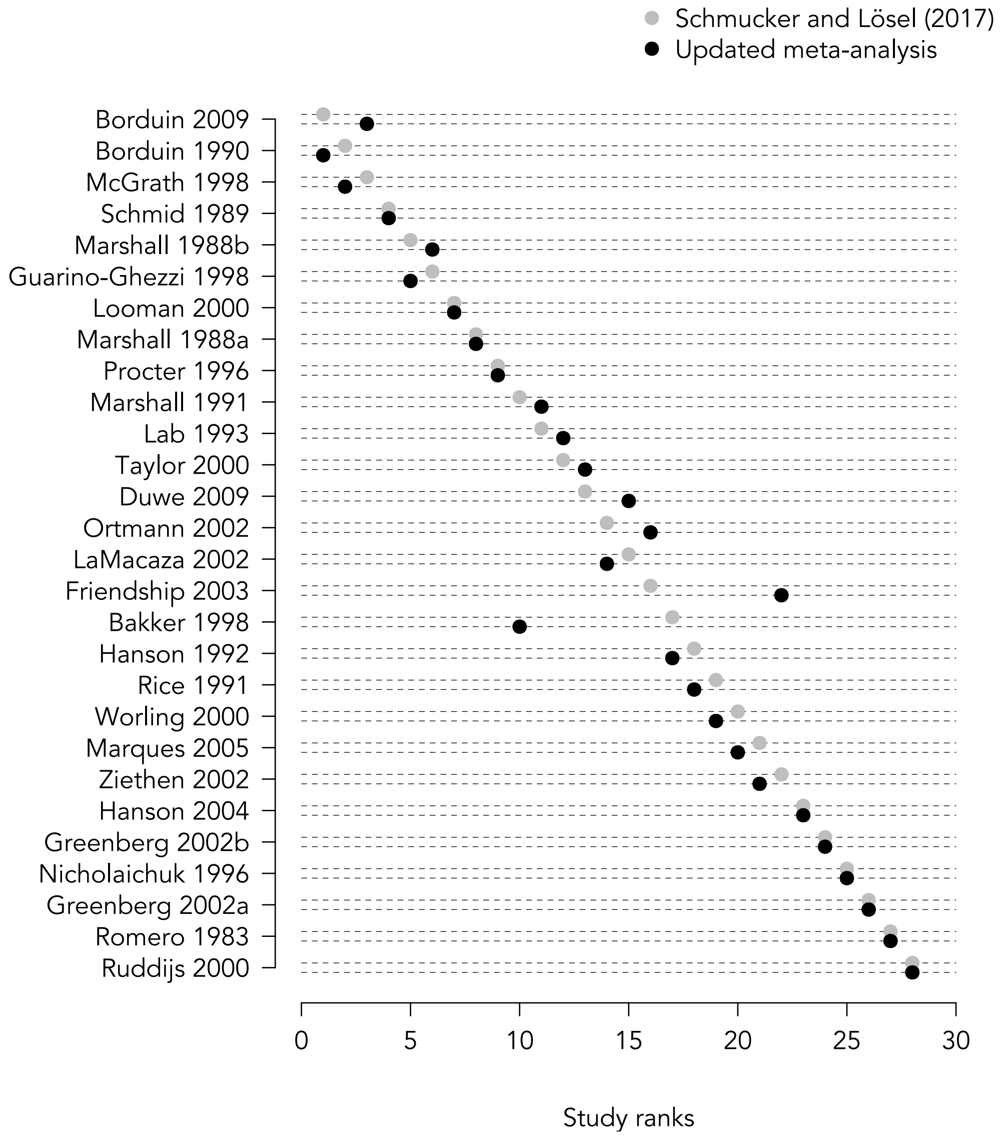

Figure 2: Dot plot. *Dot plot of the intraclass correlation coefficient (ICC) comparing the ranks of the 28 study-specific effect sizes reported by Schmucker and Lösel (2017) and that collected in the updated meta-analysis. Results indicated an excellent absolute agreement between the two ranks ($ICC_{(A,1)}$ = .971, p < .001) considering the guideline for interpreting ICC (ICC > .9 excellent) (Koo & Li, 2016).*

Meta-analysis

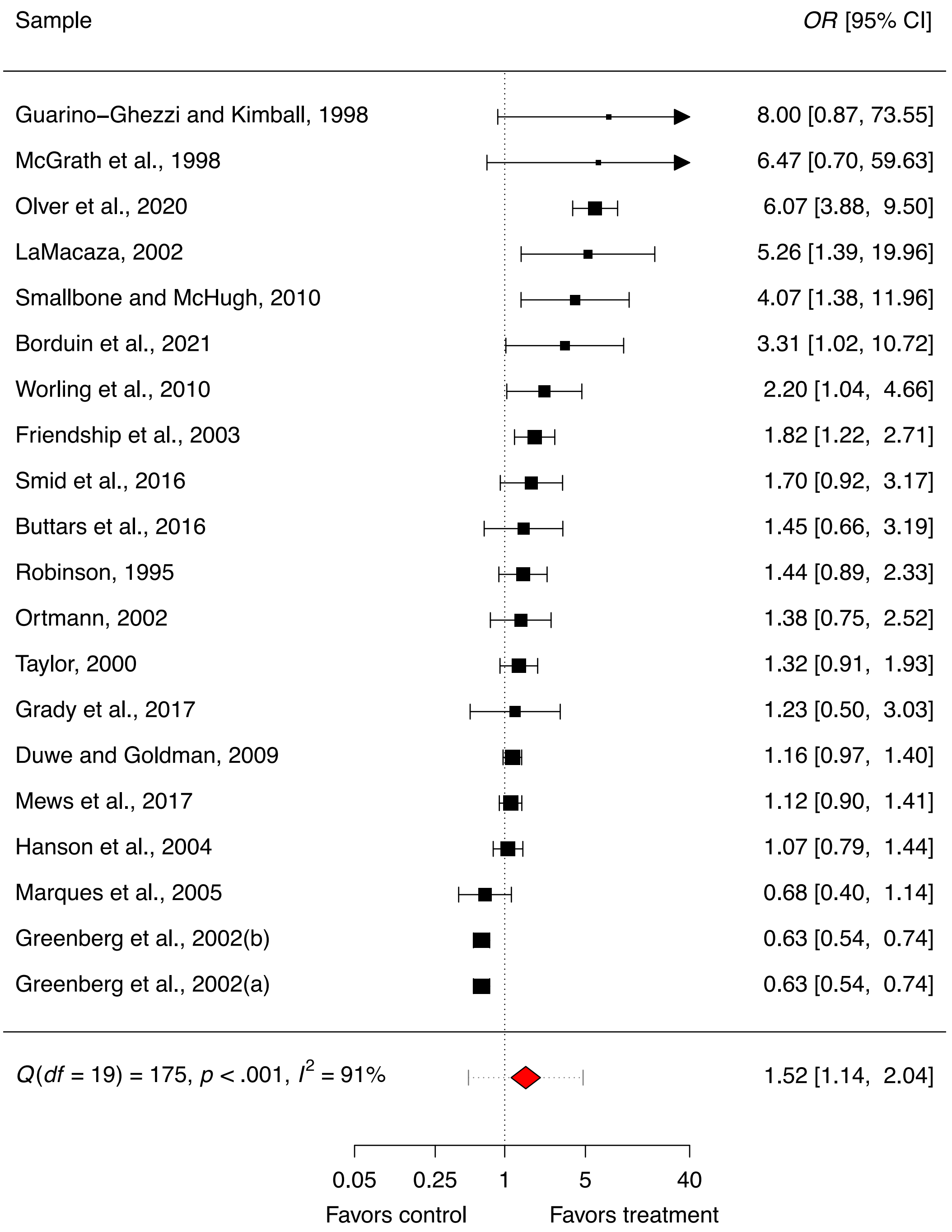

Figure 3: Forest plot violent recidivism. *Forest plot illustrating sample-specific odds ratios with 95% confidence intervals (OR [95% CI]) included in the updated meta-analysis with respect to violent recidivism. Square size is proportionate to the precision of the sample-specific effect sizes. Arrows indicate CIs extending beyond the axis limits. The red diamond represents the mean treatment effect on sexual recidivism with its 95% CIs given in brackets and its 95% prediction interval ([95% PI]) depicted as dotted interval around the diamond.*

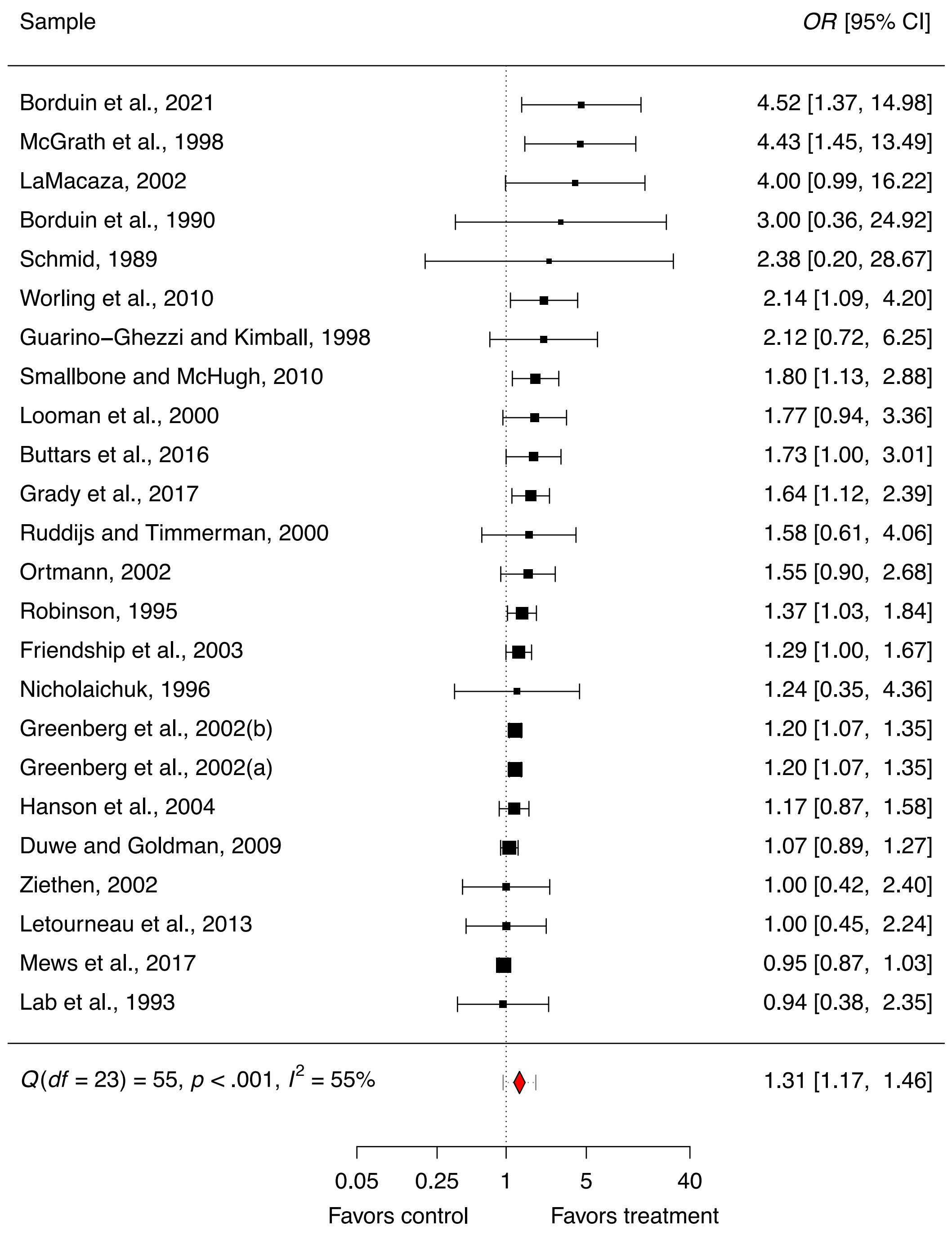

Figure 4: Forest plot general recidivism. *Forest plot illustrating sample-specific odds ratios with 95% confidence intervals (OR [95% CI]) included in the updated meta-analysis with respect to general recidivism. Square size is proportionate to the precision of the sample-specific effect sizes. Arrows indicate CIs extending beyond the axis limits. The red diamond represents the mean treatment effect on sexual recidivism with its 95% CIs given in brackets and its 95% prediction interval ([95% PI]) depicted as dotted interval around the diamond.*

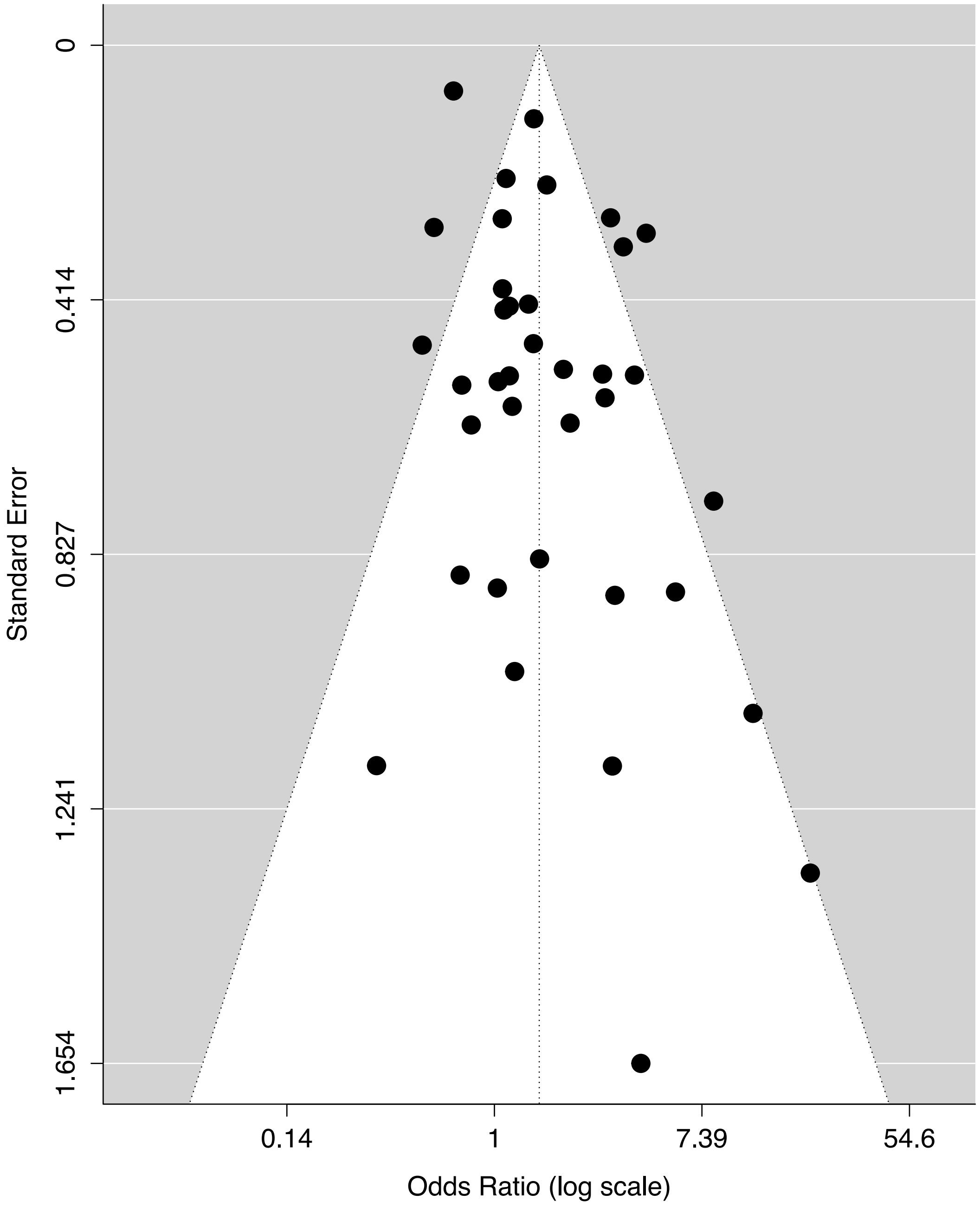

Figure 5: Funnel plot. *Funnel plot with respect to sexual recidivism. Egger's test assessing funnel plot asymmetry was not significant (z = 1.85, p = .064), thus not suggesting small-study effects.*

Moderator coding scheme

Following the coding scheme suggested by Schmucker and Lösel (2017), four types of moderators were collected, i.e., publication characteristics, sample characteristics, treatment characteristics, and individual characteristics. Together, a total of 17 moderators were collected (15 categorical predictors, 9 continuous predictors). In case of categorical moderators, subgroups are reported in brackets.

Publication characteristics

- *Publication status*: [published, unpublished]. Categorical variable.
- *Publication year*: [< 2000, ≥ 2000]. Categorical and continuous variable.
- *Country of origin*: [Canada, United States, Other]. Categorical variable.
- *Author confounding*: Author involvement in treatment program [Yes, No, Unclear]. Categorical variable.
- *Descriptive validity*: Descriptive validity refers to a report's quality in documenting the relevant details of an evaluation. Descriptive validity was coded following the concept suggested by Lösel & Köferl 1989 Lösel & Köferl, 1989. A 4-point-scale (0 = low, 1 = medium, 2 = fair, 3 = excellent) was used to judge descriptive validity in the areas of: treatment concept, treatment realization, study design, presentation of results, overall transparency of report. Continuous variable.

Sample characteristics

- *Sample size*: [< 50, 51 - 150, 151 - 250, 251 - 500, > 500]. Categorical and continuous variable.
- *Design quality*: According on the Maryland Scientific Methods Scale Farrington et al., 2002. Categorical and continuous variable.
  - Level 1: No control group (excluded from analysis).
  - Level 2: No equivalent control group (excluded from analysis).
  - Level 3: Incidental assignment of treatment and control group. The assignment strategy gave no serious doubts that assignment resulted in equivalent groups, or sound statistical control of potential differences. The assignment strategy was not related to relevant risk variables. If there was indication of potential group differences, statistical analyses must adequately take care (e.g., regression methods including relevant control variables).
  - Level 4: Matching procedures to assign treatment and control group. Systematic strategy to attain equivalence of the treatment and control group, such as theoretically sound matching or propensity score techniques. The

variables used for matching must be relevant to differences that actually or potentially arise from treatment assignment for the program under evaluation.

  - Level 5: Randomized assignment of treatment and control group. If there is attrition regarding the recidivism data, the study must be downgraded or even excluded depending on its severity. Treatment and control group must remain reasonably well comparable despite (potential) effects of selective attrition.

- *Follow-up*: [< 5 years, ≥ 5 years]. Categorical and continuous variable.
- *Recidivism definition*: [Arrest, Charge, Conviction, Multiple definitions, Unspecified]. Categorical variable.
- *Recidivism base rate*: Defined as mean recidivism rate in treatment and control group. Continuous variable.

Treatment characteristics

- *Treatment approach*: [Behavioral therapy (Laws & Marshall, 2003; Marshall & Laws, 2003), Cognitive-behavioral therapy, Insight-oriented therapy, Multisystemic therapy, Therapeutic community]. Categorical variable.
- *Treatment setting*: [Prison, Hospital, Outpatient, Mixed]. Categorical variable.
- *Treatment individualization*: [Group only, Group mainly, Mixed, Individual mainly, Individual only]. Categorical and continuous variable.
- *Treatment specialization*: [Yes, No]. Categorical variable.
- *Aftercare*: [Yes, No]. Categorical variable.

Individual characteristics

- *Age group*: [Juveniles, Adult, Mixed, Unclear]. Categorical and continuous variable.
- *Risk level*: If results of individual risk instruments (e.g., Static-99) were reported in the studies, these were used to categorize mean risk level. Otherwise, mean risk level was rated using the Rapid Risk Assessment for Sex Offence Recidivism (RRASOR Hanson, 1997). The RRASOR was originally designed for individual risk judgments. Schmucker and Lösel (2017) suggested using the RRASOR to estimate mean risk based on treatment group statistics of relevant variables according to the items: 1) Convictions for sex offenses: mean number of previous convictions for sexual offenses (max. 3); 2) Age below 25: (estimated) relative frequency of individuals aged < 25; 3) Male victims: relative frequency of offenders with male victims; 4) Any unrelated victims: relative frequency of offenders with unrelated victims. The sum of the item scores was categorized into low-risk (≤ 1.5), medium-risk (1.5 to 2.5), or high-risk (≥ 2.5). [Low-risk, Medium-risk, High-risk, Unclear]. Categorical and continuous variable.

Some other moderators examined by Schmucker and Lösel (2017) were not included in the present analysis because they were poorly documented (treatment mandate, treatment duration, treatment integrity) or unsuitably defined (offender type). None of these moderators was reported to be significantly related to sexual recidivism (Schmucker & Lösel, 2017).

Moderator sensitivity analysis

Table 1: Sensitivity analyses. *Listed are results of the sensitivity analyses of the moderator analyses with respect to sexual recidivism as indicator of treatment effectiveness. For categorical moderators, effect sizes are reported in terms of odds ratio with 95% confidence intervals (OR [95% CI]) for each subgroup. For continuous moderators (cont.), effect sizes are reported in terms of regression weights (β). An omnibus test was done for each moderator (Q_M) based on a Wald-type test, which is a test estimating whether any of the coefficients in the moderator are significantly different from zero (Viechtbauer, 2021). Significant effects of continuous moderators are underlined (p < .05). Significant subgroup-contrasts of categorical moderators are listed in the following table.*

**Moderator**	**All**	**Excluding Mews 2017**	**Excluding Borduin 1990/2021**	**Excluding Juvenile studies**	**Excluding Small samples**	**Excluding Dropouts**
	OR [95% CI]	*OR* [95% CI]	*OR* [95% CI]	*OR* [95% CI]	*OR* [95% CI]	*OR* [95% CI]
**Publication characteristics**						
**Publication status**	$Q_M(df=2)$=13,p=.001	$Q_M(df=2)$=18,p<.001	$Q_M(df=2)$=11,p=.005	$Q_M(df=2)$=9,p=.010	$Q_M(df=2)$=8,p=.017	$Q_M(df=2)$=14,p<.001
Published	1.54[1.17,2.04],p=.002	1.53[1.18,1.98],p=.001	1.44[1.09,1.89],p=.010	1.40[1.05,1.87],p=.023	1.39[1.05,1.85],p=.021	1.57[1.18,2.08],p=.002
Unpublished	1.53[1.00,2.35],p=.050	1.86[1.20,2.90],p=.006	1.52[1.01,2.30],p=.045	1.53[1.01,2.32],p=.047	1.43[0.94,2.19],p=.094	1.55[1.01,2.39],p=.045
**Publication year**	$Q_M(df=2)$=17,p<.001	$Q_M(df=2)$=20,p<.001	$Q_M(df=2)$=14,p<.001	$Q_M(df=2)$=13,p=.001	$Q_M(df=2)$=10,p=.007	$Q_M(df=2)$=18,p<.001
< 2000	2.11[1.38,3.24],p=.001	2.11[1.39,3.19],p<.001	2.00[1.31,3.05],p=.001	2.04[1.32,3.17],p=.001	1.82[1.14,2.92],p=.012	2.18[1.41,3.37],p<.001
≥ 2000	1.35[1.04,1.77],p=.025	1.44[1.11,1.88],p=.006	1.30[1.00,1.68],p=.049	1.26[0.96,1.64],p=.090	1.30[1.00,1.68],p=.052	1.37[1.05,1.79],p=.021
Publication year (cont.)	β=0.04,z=0.27,p=.787	β=0.09,z=0.66,p=.508	β=-0.00,z=-0.00,p=1.00	β=-0.02,z=-0.13,p=.895	β=0.05,z=0.35,p=.730	β=0.03,z=0.24,p=.811
**Country**	$Q_M(df=3)$=21,p<.001	$Q_M(df=3)$=24,p<.001	$Q_M(df=3)$=20,p<.001	$Q_M(df=3)$=16,p<.001	$Q_M(df=3)$=17,p<.001	$Q_M(df=3)$=21,p<.001
Canada	2.14[1.47,3.11],p<.001	2.16[1.51,3.10],p<.001	2.16[1.50,3.10],p<.001	2.10[1.43,3.07],p<.001	2.13[1.45,3.12],p<.001	2.16[1.48,3.15],p<.001
United States	1.47[0.98,2.20],p=.060	1.45[0.99,2.11],p=.055	1.24[0.83,1.84],p=.295	1.22[0.80,1.88],p=.353	1.24[0.83,1.84],p=.296	1.54[1.02,2.32],p=.040
Other	1.20[0.87,1.65],p=.260	1.34[0.96,1.85],p=.083	1.19[0.88,1.61],p=.254	1.20[0.88,1.63],p=.257	1.14[0.84,1.55],p=.390	1.20[0.87,1.66],p=.261
**Author confounding**	$Q_M(df=2)$=27,p<.001	$Q_M(df=2)$=28,p<.001	$Q_M(df=2)$=23,p<.001	$Q_M(df=2)$=21,p<.001	$Q_M(df=2)$=19,p<.001	$Q_M(df=2)$=29,p<.001
Yes	1.98[1.54,2.56],p<.001	1.98[1.54,2.56],p<.001	1.87[1.45,2.41],p<.001	1.85[1.42,2.41],p<.001	1.80[1.38,2.35],p<.001	2.02[1.56,2.61],p<.001
No	0.97[0.70,1.34],p=.858	1.05[0.74,1.50],p=.777	0.97[0.71,1.33],p=.842	0.95[0.69,1.32],p=.780	0.97[0.71,1.33],p=.847	0.98[0.71,1.35],p=.898
Unclear						
**Descriptive validity (cont.)**	β=0.55,z=3.82,p<.001	β=0.55,z=3.64,p<.001	β=0.48,z=3.63,p<.001	β=0.51,z=3.85,p<.001	β=0.48,z=3.61,p<.001	β=0.56,z=3.83,p<.001
**Sample characteristics**						
**Sample size**	$Q_M(df=5)$=22,p<.001	$Q_M(df=5)$=26,p<.001	$Q_M(df=5)$=14,p=.018	$Q_M(df=5)$=12,p=.034	$Q_M(df=4)$=9,p=.068	$Q_M(df=5)$=22,p<.001
< 50	4.62[2.01,10.62],p<.001	4.58[2.04,10.30],p<.001	3.06[1.11,8.46],p=.031	3.07[1.11,8.50],p=.031		4.62[2.01,10.64],p<.001
51 - 150	1.51[0.97,2.35],p=.071	1.50[0.98,2.31],p=.062	1.51[0.97,2.35],p=.071	1.38[0.85,2.24],p=.198	1.51[0.96,2.36],p=.073	1.51[0.97,2.37],p=.068

151 - 250	1.46[0.75,2.83],p=.267	1.48[0.79,2.77],p=.221	1.46[0.75,2.82],p=.265	1.52[0.75,3.09],p=.246	1.45[0.74,2.84],p=.277	1.54[0.78,3.03],p=.214
251 - 500	1.16[0.64,2.12],p=.628	1.16[0.66,2.04],p=.607	1.16[0.64,2.12],p=.627	1.16[0.63,2.13],p=.630	1.16[0.63,2.14],p=.632	1.17[0.64,2.15],p=.609
≥ 500	1.42[1.02,1.99],p=.040	1.60[1.14,2.23],p=.006	1.42[1.02,1.98],p=.040	1.42[1.01,1.99],p=.041	1.42[1.01,2.00],p=.043	1.44[1.03,2.02],p=.035
Sample size (cont.)	β=-0.11,z=-1.03,p=.305	β=0.02,z=0.14,p=.892	β=-0.10,z=-1.00,p=.315	β=-0.11,z=-1.02,p=.310	β=-0.10,z=-0.97,p=.334	β=-0.11,z=-1.02,p=.310
**Design quality**	$Q_M(df=3)$=18,p<.001	$Q_M(df=3)$=20,p<.001	$Q_M(df=3)$=16,p=.001	$Q_M(df=3)$=14,p=.003	$Q_M(df=3)$=12,p=.007	$Q_M(df=3)$=19,p<.001
Level 3 (incidental)	1.91[1.36,2.69],p<.001	1.91[1.37,2.66],p<.001	1.90[1.37,2.64],p<.001	1.88[1.33,2.66],p<.001	1.80[1.27,2.55],p=.001	1.95[1.38,2.75],p<.001
Level 4 (matching)	1.18[0.83,1.68],p=.353	1.31[0.90,1.89],p=.157	1.18[0.85,1.65],p=.329	1.18[0.84,1.66],p=.337	1.18[0.84,1.65],p=.334	1.19[0.84,1.70],p=.331
Level 5 (randomized)	1.62[0.93,2.80],p=.086	1.60[0.94,2.73],p=.085	1.18[0.67,2.08],p=.565	1.18[0.65,2.14],p=.587	1.18[0.67,2.09],p=.567	1.65[0.95,2.88],p=.078
Design quality (cont.)	β=-0.11,z=-0.79,p=.427	β=-0.10,z=-0.69,p=.492	β=-0.20,z=-1.50,p=.135	β=-0.19,z=-1.40,p=.162	β=-0.18,z=-1.33,p=.184	β=-0.12,z=-0.80,p=.421
**Follow-up**	$Q_M(df=2)$=14,p=.001	$Q_M(df=2)$=18,p<.001	$Q_M(df=2)$=11,p=.004	$Q_M(df=2)$=9,p=.009	$Q_M(df=2)$=9,p=.013	$Q_M(df=2)$=14,p<.001
< 5 years	1.41[0.91,2.17],p=.122	1.38[0.92,2.08],p=.122	1.31[0.85,2.01],p=.216	1.31[0.83,2.06],p=.250	1.25[0.81,1.94],p=.312	1.46[0.94,2.27],p=.094
≥ 5 years	1.59[1.21,2.10],p=.001	1.71[1.31,2.23],p<.001	1.52[1.17,1.99],p=.002	1.49[1.13,1.96],p=.005	1.47[1.12,1.93],p=.006	1.61[1.22,2.12],p=.001
Follow-up (cont.)	β=0.20,z=1.40,p=.161	β=0.22,z=1.46,p=.143	β=0.10,z=0.76,p=.445	β=0.03,z=0.22,p=.825	β=0.12,z=0.84,p=.400	β=0.20,z=1.34,p=.180
**Recidivism definition**	$Q_M(df=4)$=17,p=.002	$Q_M(df=4)$=25,p<.001	$Q_M(df=4)$=17,p=.002	$Q_M(df=4)$=15,p=.005	$Q_M(df=4)$=13,p=.011	$Q_M(df=4)$=18,p=.001
Arrest	1.26[0.85,1.87],p=.259	1.22[0.86,1.72],p=.261	1.06[0.71,1.56],p=.783	1.05[0.70,1.57],p=.806	1.06[0.71,1.57],p=.784	1.28[0.86,1.90],p=.232
Charge	1.56[0.83,2.94],p=.165	1.55[0.87,2.77],p=.136	1.56[0.85,2.88],p=.154	1.31[0.61,2.80],p=.491	1.47[0.75,2.89],p=.266	1.65[0.86,3.14],p=.129
Conviction	1.61[1.18,2.19],p=.003	1.83[1.37,2.44],p<.001	1.60[1.19,2.15],p=.002	1.60[1.19,2.16],p=.002	1.55[1.15,2.10],p=.004	1.61[1.18,2.20],p=.003
Multiple definitions						
Unspecified	2.88[1.10,7.57],p=.032	2.92[1.18,7.20],p=.020	2.90[1.13,7.43],p=.027	2.89[1.12,7.46],p=.028	2.81[0.95,8.29],p=.062	3.04[1.14,8.06],p=.026
**Recidivism base rate (cont.)**	β=0.28,z=1.98,p=.048	β=0.27,z=1.80,p=.071	β=0.20,z=1.53,p=.126	β=0.18,z=1.33,p=.184	β=0.19,z=1.44,p=.151	β=0.28,z=1.92,p=.054
**Treatment characteristics**						
**Treatment approach**	$Q_M(df=5)$=25,p<.001	$Q_M(df=5)$=30,p<.001	$Q_M(df=5)$=17,p=.004	$Q_M(df=4)$=16,p=.003	$Q_M(df=5)$=14,p=.018	$Q_M(df=5)$=26,p<.001
Behavioral therapy	2.29[1.33,3.97],p=.003	2.31[1.37,3.89],p=.002	2.29[1.33,3.97],p=.003	2.29[1.32,3.97],p=.003	2.26[1.21,4.22],p=.011	2.29[1.32,3.98],p=.003
Cognitive-behavioral therapy	1.36[1.04,1.78],p=.025	1.48[1.13,1.92],p=.004	1.36[1.04,1.78],p=.024	1.32[1.00,1.74],p=.054	1.36[1.04,1.78],p=.025	1.39[1.06,1.83],p=.018
Insight-oriented therapy	0.70[0.28,1.75],p=.447	0.70[0.29,1.67],p=.421	0.70[0.28,1.75],p=.446	0.70[0.28,1.75],p=.448	0.70[0.28,1.76],p=.449	0.70[0.28,1.76],p=.450
Multisystemic therapy	5.49[1.67,17.99],p=.005	5.49[1.72,17.55],p=.004	1.22[0.14,10.45],p=.859		1.22[0.14,10.50],p=.859	5.48[1.67,18.03],p=.005
Therapeutic community	1.58[0.93,2.67],p=.093	1.57[0.96,2.56],p=.075	1.57[0.93,2.67],p=.092	1.58[0.93,2.68],p=.093	1.42[0.81,2.47],p=.219	1.58[0.92,2.69],p=.095
**Treatment setting**	$Q_M(df=4)$=15,p=.004	$Q_M(df=4)$=19,p<.001	$Q_M(df=4)$=12,p=.019	$Q_M(df=4)$=10,p=.034	$Q_M(df=4)$=10,p=.047	$Q_M(df=4)$=16,p=.003
Prison	1.42[1.04,1.94],p=.027	1.56[1.15,2.12],p=.005	1.42[1.05,1.92],p=.022	1.41[1.04,1.91],p=.028	1.42[1.05,1.92],p=.022	1.43[1.05,1.96],p=.025
Hospital	1.28[0.72,2.27],p=.404	1.27[0.73,2.18],p=.396	1.27[0.73,2.22],p=.400	1.27[0.72,2.24],p=.402	1.11[0.62,1.99],p=.721	1.31[0.73,2.33],p=.367
Outpatient	1.92[1.23,2.99],p=.004	1.90[1.24,2.91],p=.003	1.61[1.02,2.54],p=.042	1.54[0.93,2.55],p=.093	1.50[0.91,2.46],p=.110	1.97[1.25,3.09],p=.003
Mixed	2.22[0.66,7.54],p=.199	2.24[0.69,7.31],p=.180	2.23[0.67,7.42],p=.190	2.90[0.68,12.34],p=.149	2.23[0.67,7.42],p=.189	2.34[0.67,8.21],p=.183
**Treatment individualization**	$Q_M(df=5)$=21,p<.001	$Q_M(df=5)$=25,p<.001	$Q_M(df=5)$=14,p=.016	$Q_M(df=5)$=13,p=.023	$Q_M(df=5)$=11,p=.060	$Q_M(df=5)$=22,p<.001
Group only	1.53[0.99,2.37],p=.055	1.55[1.02,2.36],p=.041	1.53[0.99,2.37],p=.053	1.53[0.99,2.37],p=.055	1.53[0.99,2.37],p=.056	1.53[0.98,2.38],p=.060
Group mainly	1.29[0.83,2.00],p=.262	1.60[0.98,2.61],p=.062	1.28[0.83,1.99],p=.263	1.27[0.81,2.01],p=.297	1.29[0.83,2.00],p=.261	1.34[0.85,2.10],p=.208
Mixed	1.39[0.98,1.99],p=.068	1.39[0.99,1.96],p=.059	1.39[0.98,1.99],p=.067	1.32[0.91,1.91],p=.139	1.33[0.92,1.91],p=.128	1.41[0.98,2.03],p=.060
Individual mainly	1.28[0.53,3.13],p=.585	1.28[0.54,3.04],p=.571	1.28[0.53,3.12],p=.583	1.28[0.53,3.13],p=.585	1.28[0.52,3.13],p=.586	1.28[0.52,3.15],p=.588

Individual only	3.68[1.81,7.51],p<.001	3.68[1.83,7.40],p<.001	2.61[1.15,5.92],p=.022	2.96[1.22,7.19],p=.016	2.79[0.88,8.80],p=.080	3.69[1.80,7.55],p<.001
Treatment individualization (cont.)	β=0.16,z=1.10,p=.271	β=0.15,z=0.99,p=.321	β=0.06,z=0.46,p=.645	β=0.08,z=0.52,p=.604	β=0.03,z=0.18,p=.856	β=0.16,z=1.08,p=.279
**Treatment specialization**	$Q_M(df=2)=19, p<.001$	$Q_M(df=2)=24, p<.001$	$Q_M(df=2)=16, p<.001$	$Q_M(df=2)=14, p<.001$	$Q_M(df=2)=13, p=.002$	$Q_M(df=2)=20, p<.001$
Yes	1.63[1.29,2.05],p<.001	1.71[1.37,2.13],p<.001	1.54[1.23,1.93],p<.001	1.52[1.20,1.93],p<.001	1.49[1.18,1.88],p=.001	1.65[1.31,2.09],p<.001
No	0.51[0.19,1.39],p=.189	0.51[0.20,1.33],p=.170	0.51[0.19,1.36],p=.181	0.51[0.19,1.37],p=.183	0.51[0.19,1.36],p=.181	0.51[0.19,1.39],p=.190
**Aftercare**	$Q_M(df=2)=14, p=.001$	$Q_M(df=2)=17, p<.001$	$Q_M(df=2)=12, p=.003$	$Q_M(df=2)=10, p=.007$	$Q_M(df=2)=9, p=.010$	$Q_M(df=2)=14, p<.001$
Yes	1.68[1.13,2.48],p=.010	1.67[1.15,2.41],p=.007	1.67[1.15,2.43],p=.008	1.65[1.13,2.41],p=.010	1.63[1.11,2.38],p=.012	1.70[1.15,2.52],p=.008
No	1.47[1.10,1.96],p=.009	1.58[1.18,2.11],p=.002	1.35[1.02,1.80],p=.035	1.32[0.98,1.78],p=.066	1.29[0.97,1.73],p=.085	1.49[1.12,1.99],p=.007
**Individual characteristics**						
**Age group**	$Q_M(df=2)=17, p<.001$	$Q_M(df=2)=21, p<.001$	$Q_M(df=2)=11, p=.004$		$Q_M(df=2)=9, p=.013$	$Q_M(df=2)=19, p<.001$
Juveniles	3.25[1.45,7.29],p=.004	3.25[1.48,7.14],p=.003	1.94[0.74,5.08],p=.177		1.94[0.74,5.08],p=.178	3.71[1.62,8.50],p=.002
Adults	1.44[1.14,1.82],p=.002	1.51[1.20,1.90],p<.001	1.44[1.14,1.81],p=.002		1.38[1.08,1.75],p=.009	1.45[1.15,1.83],p=.002
Mixed						
Unclear						
Age group (cont.)	β=-0.04,z=-0.26,p=.798	β=0.01,z=0.06,p=.951	β=0.10,z=0.66,p=.512	β=0.12,z=0.84,p=.402	β=0.11,z=0.65,p=.513	β=-0.06,z=-0.35,p=.724
**Risk level**	$Q_M(df=4)=110, p<.001$	$Q_M(df=4)=57, p<.001$	$Q_M(df=4)=99, p<.001$	$Q_M(df=4)=95, p<.001$	$Q_M(df=4)=93, p<.001$	$Q_M(df=4)=113, p<.001$
Low-risk	0.68[0.60,0.78],p<.001	0.74[0.50,1.10],p=.140	0.68[0.60,0.78],p<.001	0.68[0.60,0.78],p<.001	0.68[0.60,0.78],p<.001	0.68[0.60,0.78],p<.001
Medium-risk	1.57[1.35,1.83],p<.001	1.63[1.30,2.04],p<.001	1.57[1.35,1.83],p<.001	1.58[1.35,1.84],p<.001	1.56[1.34,1.82],p<.001	1.65[1.40,1.95],p<.001
High-risk	4.26[2.67,6.78],p<.001	4.47[2.63,7.57],p<.001	3.71[2.25,6.13],p<.001	4.02[2.25,7.17],p<.001	3.57[2.12,6.02],p<.001	4.24[2.66,6.76],p<.001
Unspecified	1.69[1.19,2.41],p=.004	1.70[1.09,2.66],p=.020	1.69[1.19,2.41],p=.004	1.69[1.19,2.41],p=.004	1.66[1.15,2.40],p=.007	1.70[1.19,2.42],p=.004
Risk level (cont.)	β=0.33,z=2.62,p=.009	β=0.31,z=2.32,p=.020	β=0.31,z=2.50,p=.012	β=0.31,z=2.41,p=.016	β=0.31,z=2.48,p=.013	β=0.33,z=2.58,p=.010

Table 2: Sensitivity analyses subgroup-contrasts. *Listed are contrasts of the categorical moderator subgroups based on general linear hypothesis testing. The Bonferroni correction was applied to counteract the problem of multiple comparisons. Significant subgroup differences are underlined (p < .05).*

Moderator	Subgroup-contrast	All	Excluding Mews 2017	Excluding Borduin 1990/2021	Excluding Juvenile studies	Excluding Small samples	Excluding Dropouts
**Publication characteristics**							
Publication status	Published : Unpublished	z=-0.02,p=.981	z=0.75,p=.454	z=0.23,p=.815	z=0.34,p=.736	z=0.11,p=.915	z=-0.04,p=.969
Publication year	< 2000 : ≥ 2000	z=-1.73,p=.083	z=-1.51,p=.130	z=-1.71,p=.087	z=-1.85,p=.065	z=-1.24,p=.213	z=-1.79,p=.073
Country	Canada : Other	z=-2.30,p=.056	z=-1.94,p=.127	z=-2.47,p=.036	z=-2.23,p=.066	z=-2.48,p=.034	z=-2.32,p=.053
Country	Canada : United States	z=-1.33,p=.376	z=-1.51,p=.285	z=-2.03,p=.103	z=-1.83,p=.158	z=-1.93,p=.129	z=-1.20,p=.455
Country	Other : United States	z=0.78,p=.718	z=0.31,p=.948	z=0.14,p=.989	z=0.09,p=.996	z=0.30,p=.951	z=0.93,p=.623
Author confounding	No : Yes	z=3.40,p=.001	z=2.83,p=.005	z=3.19,p=.001	z=3.09,p=.002	z=2.94,p=.003	z=3.44,p=.001
**Sample characteristics**							
Sample size	151 - 250 : 251 - 500	z=-0.50,p=.987	z=-0.57,p=.979	z=-0.50,p=.987	z=-0.57,p=.978	z=-0.48,p=.962	z=-0.58,p=.976
Sample size	151 - 250 : 51 - 150	z=0.08,p=1.00	z=0.04,p=1.00	z=0.08,p=1.00	z=-0.23,p=.999	z=0.09,p=1.00	z=-0.04,p=1.00
Sample size	251 - 500 : 51 - 150	z=0.68,p=.958	z=0.72,p=.950	z=0.68,p=.957	z=0.43,p=.992	z=0.67,p=.904	z=0.67,p=.962
Sample size	< 50 : 151 - 250	z=-2.13,p=.200	z=-2.16,p=.187	z=-1.20,p=.737	z=-1.11,p=.793		z=-2.01,p=.252
Sample size	< 50 : 251 - 500	z=-2.63,p=.061	z=-2.72,p=.048	z=-1.61,p=.472	z=-1.60,p=.478		z=-2.60,p=.066
Sample size	< 50 : 51 - 150	z=-2.33,p=.129	z=-2.38,p=.114	z=-1.26,p=.704	z=-1.39,p=.620		z=-2.31,p=.134
Sample size	< 50 : ≥ 500	z=-2.57,p=.071	z=-2.35,p=.122	z=-1.41,p=.605	z=-1.40,p=.611		z=-2.54,p=.077
Sample size	≥ 500 : 151 - 250	z=0.06,p=1.00	z=-0.21,p=1.00	z=0.07,p=1.00	z=0.17,p=1.00	z=0.05,p=1.00	z=0.17,p=1.00
Sample size	≥ 500 : 251 - 500	z=-0.57,p=.978	z=-0.96,p=.868	z=-0.58,p=.977	z=-0.57,p=.978	z=-0.57,p=.939	z=-0.57,p=.977
Sample size	≥ 500 : 51 - 150	z=0.21,p=1.00	z=-0.22,p=.999	z=0.21,p=1.00	z=-0.10,p=1.00	z=0.20,p=.997	z=0.18,p=1.00
Design quality	Level 3 : Level 4	z=-1.93,p=.128	z=-1.49,p=.291	z=-2.01,p=.107	z=-1.87,p=.143	z=-1.71,p=.199	z=-1.96,p=.120
Design quality	Level 3 : Level 5	z=-0.51,p=.866	z=-0.55,p=.847	z=-1.43,p=.319	z=-1.32,p=.378	z=-1.23,p=.428	z=-0.50,p=.868
Design quality	Level 4 : Level 5	z=0.95,p=.608	z=0.61,p=.812	z=0.00,p=1.00	z=-0.00,p=1.00	z=0.00,p=1.00	z=0.97,p=.594
Follow up	< 5 years : ≥ 5 years	z=0.48,p=.634	z=0.86,p=.387	z=0.59,p=.553	z=0.48,p=.628	z=0.61,p=.542	z=0.37,p=.714
Recidivism definition	Arrest : Charge	z=0.58,p=.935	z=0.70,p=.889	z=1.05,p=.706	z=0.49,p=.957	z=0.82,p=.833	z=0.66,p=.906
Recidivism definition	Arrest : Conviction	z=0.96,p=.760	z=1.76,p=.279	z=1.66,p=.329	z=1.65,p=.333	z=1.52,p=.405	z=0.90,p=.793
Recidivism definition	Arrest : Unspecified	z=1.56,p=.384	z=1.77,p=.272	z=1.93,p=.200	z=1.93,p=.202	z=1.66,p=.325	z=1.61,p=.356
Recidivism definition	Charge : Conviction	z=0.08,p=1.00	z=0.49,p=.959	z=0.08,p=1.00	z=0.49,p=.959	z=0.15,p=.999	z=-0.06,p=1.00
Recidivism definition	Charge : Unspecified	z=1.04,p=.713	z=1.15,p=.641	z=1.08,p=.689	z=1.28,p=.556	z=0.99,p=.738	z=1.02,p=.723
Recidivism definition	Conviction : Unspecified	z=1.13,p=.657	z=0.97,p=.754	z=1.17,p=.627	z=1.17,p=.631	z=1.03,p=.716	z=1.21,p=.605
**Treatment characteristics**							
Treatment approach	Behavioral : Cognitive-behavioral	z=-1.68,p=.422	z=-1.51,p=.532	z=-1.68,p=.407	z=-1.77,p=.274	z=-1.45,p=.558	z=-1.59,p=.478

Treatment approach	Behavioral : Insight-oriented	z=-2.18,p=.171	z=-2.31,p=.127	z=-2.18,p=.162	z=-2.17,p=.123	z=-2.06,p=.210	z=-2.16,p=.176
Treatment approach	Behavioral : Multisystemic	z=1.31,p=.664	z=1.33,p=.647	z=-0.56,p=.977		z=-0.54,p=.980	z=1.30,p=.667
Treatment approach	Behavioral : Therapeutic community	z=-0.97,p=.857	z=-1.07,p=.808	z=-0.97,p=.850	z=-0.96,p=.759	z=-1.09,p=.787	z=-0.96,p=.862
Treatment approach	Cognitive-behavioral : Insight-oriented	z=-1.36,p=.628	z=-1.61,p=.464	z=-1.36,p=.615	z=-1.29,p=.556	z=-1.36,p=.621	z=-1.40,p=.603
Treatment approach	Cognitive-behavioral : Multisystemic	z=2.24,p=.148	z=2.16,p=.176	z=-0.10,p=1.00		z=-0.10,p=1.00	z=2.20,p=.163
Treatment approach	Cognitive-behavioral : Therapeutic community	z=0.48,p=.987	z=0.21,p=1.00	z=0.48,p=.987	z=0.59,p=.931	z=0.12,p=1.00	z=0.41,p=.994
Treatment approach	Insight-oriented : Multisystemic	z=2.69,p=.049	z=2.78,p=.038	z=0.46,p=.989		z=0.46,p=.989	z=2.68,p=.051
Treatment approach	Insight-oriented : Therapeutic community	z=1.50,p=.536	z=1.58,p=.484	z=1.50,p=.523	z=1.50,p=.423	z=1.28,p=.670	z=1.49,p=.542
Treatment approach	Multisystemic : Therapeutic community	z=-1.88,p=.304	z=-1.95,p=.268	z=0.23,p=.999		z=0.13,p=1.00	z=-1.87,p=.308
Treatment setting	Hospital : Mixed	z=0.81,p=.841	z=0.86,p=.812	z=0.83,p=.827	z=1.04,p=.706	z=1.02,p=.720	z=0.83,p=.829
Treatment setting	Hospital : Outpatient	z=1.10,p=.673	z=1.15,p=.641	z=0.64,p=.913	z=0.49,p=.957	z=0.76,p=.862	z=1.09,p=.675
Treatment setting	Hospital : Prison	z=0.32,p=.988	z=0.65,p=.909	z=0.34,p=.985	z=0.31,p=.989	z=0.73,p=.877	z=0.27,p=.992
Treatment setting	Mixed : Outpatient	z=-0.22,p=.996	z=-0.26,p=.993	z=-0.50,p=.956	z=-0.81,p=.837	z=-0.60,p=.926	z=-0.26,p=.993
Treatment setting	Mixed : Prison	z=-0.70,p=.890	z=-0.59,p=.932	z=-0.72,p=.882	z=-0.96,p=.755	z=-0.72,p=.882	z=-0.75,p=.868
Treatment setting	Outpatient : Prison	z=-1.08,p=.683	z=-0.74,p=.874	z=-0.45,p=.967	z=-0.30,p=.990	z=-0.18,p=.998	z=-1.14,p=.646
Treatment individualization	Group mainly : Group only	z=0.55,p=.980	z=-0.09,p=1.00	z=0.57,p=.978	z=0.57,p=.977	z=0.55,p=.981	z=0.41,p=.994
Treatment individualization	Group mainly : Individual mainly	z=-0.01,p=1.00	z=-0.43,p=.992	z=-0.00,p=1.00	z=0.01,p=1.00	z=-0.01,p=1.00	z=-0.08,p=1.00
Treatment individualization	Group mainly : Individual only	z=2.46,p=.093	z=1.91,p=.298	z=1.50,p=.548	z=1.66,p=.442	z=1.23,p=.714	z=2.34,p=.124
Treatment individualization	Group mainly : Mixed	z=0.28,p=.999	z=-0.45,p=.991	z=0.29,p=.998	z=0.12,p=1.00	z=0.10,p=1.00	z=0.19,p=1.00
Treatment individualization	Group only : Individual mainly	z=-0.35,p=.996	z=-0.39,p=.995	z=-0.36,p=.996	z=-0.35,p=.996	z=-0.35,p=.996	z=-0.34,p=.997
Treatment individualization	Group only : Individual only	z=2.06,p=.228	z=2.08,p=.219	z=1.12,p=.784	z=1.31,p=.672	z=0.96,p=.864	z=2.05,p=.231
Treatment individualization	Group only : Mixed	z=-0.33,p=.997	z=-0.39,p=.995	z=-0.33,p=.997	z=-0.51,p=.986	z=-0.49,p=.987	z=-0.26,p=.999
Treatment individualization	Individual mainly : Individual only	z=1.81,p=.351	z=1.86,p=.327	z=1.15,p=.766	z=1.31,p=.672	z=1.05,p=.820	z=1.80,p=.359
Treatment individualization	Individual mainly : Mixed	z=0.17,p=1.00	z=0.17,p=1.00	z=0.17,p=1.00	z=0.06,p=1.00	z=0.07,p=1.00	z=0.20,p=1.00
Treatment individualization	Individual only : Mixed	z=-2.39,p=.111	z=-2.44,p=.098	z=-1.38,p=.628	z=-1.65,p=.450	z=-1.21,p=.729	z=-2.34,p=.125
Treatment specialization	No : Yes	z=2.21,p=.027	z=2.40,p=.016	z=2.15,p=.032	z=2.11,p=.035	z=2.07,p=.038	z=2.24,p=.025
Aftercare	No : Yes	z=0.53,p=.597	z=0.22,p=.823	z=0.87,p=.386	z=0.90,p=.368	z=0.94,p=.346	z=0.53,p=.593
**Individual characteristics**							
Age group	Adults : Juveniles	z=1.91,p=.057	z=1.83,p=.067	z=0.59,p=.553		z=0.67,p=.500	z=2.13,p=.033
Risk level	High-risk : Low-risk	z=-7.40, p<.001	z=-5.33, p<.001	z=-6.40, p<.001	z=-5.84, p<.001	z=-6.00, p<.001	z=-7.38, p<.001
Risk level	High-risk : Medium-risk	z=-3.98, p<.001	z=-3.44,p=.003	z=-3.22,p=.006	z=-3.05,p=.010	z=-2.97,p=.013	z=-3.74,p=.001
Risk level	High-risk : Unspecified	z=-3.09,p=.009	z=-2.74,p=.030	z=-2.51,p=.051	z=-2.50,p=.052	z=-2.34,p=.078	z=-3.06,p=.010
Risk level	Low-risk : Medium-risk	z=8.00, p<.001	z=3.39,p=.003	z=8.00, p<.001	z=8.01, p<.001	z=7.93, p<.001	z=8.19, p<.001
Risk level	Low-risk : Unspecified	z=4.70, p<.001	z=2.72,p=.031	z=4.70, p<.001	z=4.70, p<.001	z=4.42, p<.001	z=4.69, p<.001
Risk level	Medium-risk : Unspecified	z=0.38,p=.980	z=0.16,p=.998	z=0.38,p=.980	z=0.35,p=.983	z=0.30,p=.990	z=0.13,p=.999

Table 3: Sensitivity analyses heterogeneity. *Listed are Q-values indicating residual heterogeneity after accounting for each moderator based on the moderator analyses with respect to sexual recidivism as an indicator of treatment effectiveness.*

**Moderator**	**All**	**Excluding Mews 2017**	**Excluding Borduin 1990/2021**	**Excluding Juvenile studies**	**Excluding Small samples**	**Excluding Dropouts**
Publication status	$Q(df{=}35){=}116, p{<}.001$	$Q(df{=}34){=}72, p{<}.001$	$Q(df{=}33){=}106, p{<}.001$	$Q(df{=}29){=}104, p{<}.001$	$Q(df{=}30){=}100, p{<}.001$	$Q(df{=}35){=}115, p{<}.001$
Publication year (cont.)	$Q(df{=}36){=}141, p{<}.001$	$Q(df{=}35){=}109, p{<}.001$	$Q(df{=}34){=}126, p{<}.001$	$Q(df{=}30){=}121, p{<}.001$	$Q(df{=}31){=}119, p{<}.001$	$Q(df{=}36){=}142, p{<}.001$
Publication year	$Q(df{=}35){=}125, p{<}.001$	$Q(df{=}34){=}70, p{<}.001$	$Q(df{=}33){=}114, p{<}.001$	$Q(df{=}29){=}109, p{<}.001$	$Q(df{=}30){=}112, p{<}.001$	$Q(df{=}35){=}126, p{<}.001$
Country	$Q(df{=}34){=}91, p{<}.001$	$Q(df{=}33){=}63, p{=}.001$	$Q(df{=}32){=}81, p{<}.001$	$Q(df{=}28){=}80, p{<}.001$	$Q(df{=}29){=}76, p{<}.001$	$Q(df{=}34){=}90, p{<}.001$
Author confounding	$Q(df{=}35){=}77, p{<}.001$	$Q(df{=}34){=}65, p{<}.001$	$Q(df{=}33){=}69, p{<}.001$	$Q(df{=}29){=}66, p{<}.001$	$Q(df{=}30){=}66, p{<}.001$	$Q(df{=}35){=}74, p{<}.001$
Descriptive validity (cont.)	$Q(df{=}36){=}117, p{<}.001$	$Q(df{=}35){=}109, p{<}.001$	$Q(df{=}34){=}103, p{<}.001$	$Q(df{=}30){=}95, p{<}.001$	$Q(df{=}31){=}95, p{<}.001$	$Q(df{=}36){=}116, p{<}.001$
Sample size (cont.)	$Q(df{=}36){=}121, p{<}.001$	$Q(df{=}35){=}111, p{<}.001$	$Q(df{=}34){=}107, p{<}.001$	$Q(df{=}30){=}103, p{<}.001$	$Q(df{=}31){=}100, p{<}.001$	$Q(df{=}36){=}122, p{<}.001$
Sample size	$Q(df{=}32){=}121, p{<}.001$	$Q(df{=}31){=}64, p{<}.001$	$Q(df{=}30){=}118, p{<}.001$	$Q(df{=}26){=}116, p{<}.001$	$Q(df{=}28){=}117, p{<}.001$	$Q(df{=}32){=}121, p{<}.001$
Design quality (cont.)	$Q(df{=}36){=}140, p{<}.001$	$Q(df{=}35){=}119, p{<}.001$	$Q(df{=}34){=}124, p{<}.001$	$Q(df{=}30){=}120, p{<}.001$	$Q(df{=}31){=}117, p{<}.001$	$Q(df{=}36){=}140, p{<}.001$
Design quality	$Q(df{=}34){=}111, p{<}.001$	$Q(df{=}33){=}71, p{<}.001$	$Q(df{=}32){=}100, p{<}.001$	$Q(df{=}28){=}98, p{<}.001$	$Q(df{=}29){=}98, p{<}.001$	$Q(df{=}34){=}110, p{<}.001$
Follow up (cont.)	$Q(df{=}36){=}144, p{<}.001$	$Q(df{=}35){=}103, p{<}.001$	$Q(df{=}34){=}134, p{<}.001$	$Q(df{=}30){=}130, p{<}.001$	$Q(df{=}31){=}126, p{<}.001$	$Q(df{=}36){=}146, p{<}.001$
Follow up	$Q(df{=}35){=}145, p{<}.001$	$Q(df{=}34){=}71, p{<}.001$	$Q(df{=}33){=}133, p{<}.001$	$Q(df{=}29){=}129, p{<}.001$	$Q(df{=}30){=}127, p{<}.001$	$Q(df{=}35){=}147, p{<}.001$
Recidivism definition	$Q(df{=}33){=}131, p{<}.001$	$Q(df{=}32){=}64, p{<}.001$	$Q(df{=}31){=}120, p{<}.001$	$Q(df{=}27){=}118, p{<}.001$	$Q(df{=}28){=}116, p{<}.001$	$Q(df{=}33){=}131, p{<}.001$
Recidivism base rate (cont.)	$Q(df{=}36){=}117, p{<}.001$	$Q(df{=}35){=}111, p{<}.001$	$Q(df{=}34){=}106, p{<}.001$	$Q(df{=}30){=}104, p{<}.001$	$Q(df{=}31){=}98, p{<}.001$	$Q(df{=}36){=}119, p{<}.001$
Treatment approach	$Q(df{=}32){=}112, p{<}.001$	$Q(df{=}31){=}60, p{=}.002$	$Q(df{=}30){=}108, p{<}.001$	$Q(df{=}27){=}104, p{<}.001$	$Q(df{=}27){=}106, p{<}.001$	$Q(df{=}32){=}113, p{<}.001$
Treatment setting	$Q(df{=}33){=}134, p{<}.001$	$Q(df{=}32){=}71, p{<}.001$	$Q(df{=}31){=}125, p{<}.001$	$Q(df{=}27){=}122, p{<}.001$	$Q(df{=}28){=}121, p{<}.001$	$Q(df{=}33){=}134, p{<}.001$
Treatment individualization (cont.)	$Q(df{=}36){=}145, p{<}.001$	$Q(df{=}35){=}119, p{<}.001$	$Q(df{=}34){=}133, p{<}.001$	$Q(df{=}30){=}128, p{<}.001$	$Q(df{=}31){=}127, p{<}.001$	$Q(df{=}36){=}147, p{<}.001$
Treatment individualization	$Q(df{=}32){=}87, p{<}.001$	$Q(df{=}31){=}65, p{<}.001$	$Q(df{=}30){=}84, p{<}.001$	$Q(df{=}26){=}80, p{<}.001$	$Q(df{=}27){=}81, p{<}.001$	$Q(df{=}32){=}88, p{<}.001$
Treatment specialization	$Q(df{=}35){=}142, p{<}.001$	$Q(df{=}34){=}68, p{<}.001$	$Q(df{=}33){=}130, p{<}.001$	$Q(df{=}29){=}126, p{<}.001$	$Q(df{=}30){=}124, p{<}.001$	$Q(df{=}35){=}144, p{<}.001$
Aftercare	$Q(df{=}35){=}123, p{<}.001$	$Q(df{=}34){=}75, p{<}.001$	$Q(df{=}33){=}108, p{<}.001$	$Q(df{=}29){=}103, p{<}.001$	$Q(df{=}30){=}102, p{<}.001$	$Q(df{=}35){=}122, p{<}.001$
Age group (cont.)	$Q(df{=}33){=}135, p{<}.001$	$Q(df{=}32){=}103, p{<}.001$	$Q(df{=}31){=}122, p{<}.001$	$Q(df{=}27){=}114, p{<}.001$	$Q(df{=}28){=}115, p{<}.001$	$Q(df{=}33){=}135, p{<}.001$
Age group	$Q(df{=}35){=}136, p{<}.001$	$Q(df{=}34){=}71, p{<}.001$	$Q(df{=}33){=}131, p{<}.001$		$Q(df{=}30){=}124, p{<}.001$	$Q(df{=}35){=}135, p{<}.001$
Risk level (cont.)	$Q(df{=}36){=}110, p{<}.001$	$Q(df{=}35){=}111, p{<}.001$	$Q(df{=}34){=}96, p{<}.001$	$Q(df{=}30){=}91, p{<}.001$	$Q(df{=}31){=}87, p{<}.001$	$Q(df{=}36){=}111, p{<}.001$
Risk level	$Q(df{=}33){=}39, p{=}.227$	$Q(df{=}32){=}39, p{=}.198$	$Q(df{=}31){=}36, p{=}.238$	$Q(df{=}27){=}36, p{=}.124$	$Q(df{=}28){=}35, p{=}.164$	$Q(df{=}33){=}37, p{=}.282$

References

References marked with an asterisk indicate the studies included in the meta-analysis.

* Abracen, J., Looman, J., Ferguson, M., Harkins, L., & Mailloux, D. (2011). Recidivism among treated sexual offenders and comparison subjects: Recent outcome data from the Regional Treatment Centre (Ontario) high-intensity Sex Offender Treatment Programme. *Journal of Sexual Aggression*, *17*(2), 142–152. https://doi.org/10.1080/13552600903511980

* Bakker, L., Hudson, S., Wales, D., & Riley, D. (1998). *And there was light: Evaluating the Kia Marama treatment programme for New Zealand sex offenders against children*. Department of Corrections.

* Borduin, C. M., Henggeler, S., Blaske, D., & Stein, R. (1990). Multisystemic Treatment of Adolescent Sexual Offenders. *International Journal of Offender Therapy and Comparative Criminology*, *34*, 105–113. https://doi.org/10.1177/0306624X9003400204

Borduin, C. M., Quetsch, L. B., Johnides, B. D., & Dopp, A. R. (2021). Long-term effects of multisystemic therapy for problem sexual behaviors: A 24.9-year follow-up to a randomized clinical trial. *Journal of Consulting and Clinical Psychology*, *89*(5), 393–405. https://doi.org/10.1037/ccp0000646

Borduin, C. M., Schaeffer, C., & Heiblum, N. (2009). A Randomized Clinical Trial of Multisystemic Therapy With Juvenile Sexual Offenders: Effects on Youth Social Ecology and Criminal Activity. *Journal of Consulting and Clinical Psychology*, *77*, 26–37. https://doi.org/10.1037/a0013035

* Buttars, A., Huss, M. T., & Brack, C. (2016). An analysis of an intensive supervision program for sex offenders using propensity scores. *Journal of Offender Rehabilitation*, *55*(1), 51–68. https://doi.org/10.1080/10509674.2015.1107003

* Duwe, G., & Goldman, R. A. (2009). The Impact of Prison-Based Treatment on Sex Offender Recidivism:Evidence From Minnesota. *Sexual Abuse*, *21*(3), 279–307. https://doi.org/10.1177/1079063209338490

Farrington, DP., Gottfredson, DC., Sherman, LW., & Welsh, BC. (2002). The Maryland Scientific Methods Scale. In LW. Sherman, DP. Farrington, BC. Welsh, & DL. MacKenzie (Eds.), *Evidence-based crime prevention* (pp. 13–21). Routledge.

* Friendship, C., Mann, R. E., & Beech, A. R. (2003). Evaluation of a National Prison-based Treatment Program for Sexual Offenders in England and Wales. *Journal of Interpersonal Violence*, *18*(7), 744–759. https://doi.org/10.1177/0886260503253236

Gannon, T. A., Olver, M. E., Mallion, J. S., & James, M. (2019). Does specialized psychological treatment for offending reduce recidivism? A meta-analysis examining staff and program variables as predictors of treatment effectiveness. *Clinical Psychology Review*, *73*, 101752. https://doi.org/https://doi.org/10.1016/j.cpr.2019.101752

* Grady, M. D., Edwards, Jr. D., & Pettus-Davis, C. (2017). A Longitudinal Outcome Evaluation of a Prison-Based Sex Offender Treatment Program. *Sexual Abuse*, *29*(3), 239–266. https://doi.org/10.1177/1079063215585731

* Greenberg, D. M., daSilva, JA., & Loh, N. (2002). *Evaluation of The Western Australian Sex Offender Treatment Unit (1987-1999): A Quantitative Analysis*. Forensic Research Unit, Department of Psychiatry; Behavioural Sciences & Crime Research Centre: University of Western Australia.

* Guarino-Ghezzi, S., & Kimball, L. M. (1998). Juvenile sex offenders in treatment. *Corrections Management Quarterly*, *2*(1), 45–54.

Haddaway, N., McGuinness, L., & Pritchard, C. (2021). *Package "PRISMA2020." Make Interactive 'PRISMA' Flow Diagrams*.

Hanson, R. K. (1997). *The development of a brief actuarial risk scale for sexual offense recidivism. (User Report 1997-04)*. Department of the Solicitor General of Canada.

* Hanson, R. K., Broom, I., & Stephenson, M. (2004). Evaluating Community Sex Offender Treatment Programs: A 12-Year Follow-Up of 724 Offenders. *Canadian Journal of Behavioural Science/Revue Canadienne Des Sciences Du Comportement*, *36*, 87–96. https://doi.org/10.1037/h0087220

* Hanson, R. K., Steffy, R. A., & Gauthier, R. (1992). *Long-term follow-up of child molesters: Risk prediction and treatment outcome. (User Report No. 1992-02).* Corrections Branch, Ministry of the Solicitor General of Canada.

Koo, T. K., & Li, M. Y. (2016). A Guideline of Selecting and Reporting Intraclass Correlation Coefficients for Reliability Research. *Journal of Chiropractic Medicine*, *15*(2), 155–163. https://doi.org/https://doi.org/10.1016/j.jcm.2016.02.012

* Lab, S. P., Shields, G., & Schondel, C. (1993). Research Note: An Evaluation of Juvenile Sexual Offender Treatment. *Crime & Delinquency*, *39*(4), 543–553. https://doi.org/10.1177/0011128793039004008

* LaMacaza. (2002). *Criterion 8 - Program Follow-up and ongoing assessment*.

Laws, D. R., & Marshall, W. L. (2003). A Brief History of Behavioral and Cognitive Behavioral Approaches to Sexual Offenders: Part 1. Early Developments. *Sexual Abuse*, *15*(2), 75–92. https://doi.org/10.1177/107906320301500201

* Letourneau, E. J., Henggeler, S. W., McCart, M. R., Borduin, C. M., Schewe, P. A., & Armstrong, K. S. (2013). Two-year follow-up of a randomized effectiveness trial evaluating MST for juveniles who sexually offend. *Journal of Family Psychology*, *27*(6), 978–985. https://doi.org/10.1037/a0034710

* Looman, J., Abracen, J., & Nicholaichuk, T. (2000). Recidivism Among Treated Sexual Offenders and Matched ControlsData From the Regional Treatment Centre (Ontario). *Journal of Interpersonal Violence*, *15*, 279–290. https://doi.org/10.1177/088626000015003004

Lösel, F. (2020). Entwicklungspfade der Straftäterbehandlung: skizzierte Wege und Evaluation der Zielerreichung. *Forensische Psychiatrie, Psychologie, Kriminologie*, *14*(1), 35–49. https://doi.org/10.1007/s11757-020-00582-4

Lösel, F., & Köferl, P. (1989). Evaluation research on correctional treatment in West Germany: A meta-analysis. In H. Wegener, F. Lösel, & J. Haisch (Eds.), *Criminal behavior and the justice system: Psychological perspectives*. Springer.

* Marques, J. K., Wiederanders, M., Day, D. M., Nelson, C., & Ommeren, A. van. (2005). Effects of a relapse prevention program on sexual recidivism: final results from California's sex offender treatment and evaluation project (SOTEP). *Sexual Abuse*, *17*(1), 79–107. https://doi.org/10.1177/107906320501700108

* Marshall, W. L., & Barbaree, H. E. (1988). The long-term evaluation of a behavioral treatment program for child molesters. *Behaviour Research and Therapy*, *26*(6), 499–511. https://doi.org/https://doi.org/10.1016/0005-7967(88)90146-5

* Marshall, W. L., Eccles, A., & Barbaree, H. E. (1991). The treatment of exhibitionists: A focus on sexual deviance versus cognitive and relationship features. *Behaviour Research and Therapy*, *29*(2), 129–135. https://doi.org/https://doi.org/10.1016/0005-7967(91)90041-Z

Marshall, W. L., & Laws, D. R. (2003). A brief history of behavioral and cognitive behavioral approaches to sexual offender treatment: Part 2. The modern era. *Sexual Abuse*, *15*(2), 93–120. https://doi.org/10.1177/107906320301500202

* McGrath, R., Hoke, S., & Vojtisek, J. (1998). Cognitive-Behavioral Treatment of Sex Offenders: A Treatment Comparison and Long-Term Follow-Up Study. *Criminal Justice and Behavior*, *25*, 203–225. https://doi.org/10.1177/0093854898025002004

* Mews, A., Bella, L., & Purver, M. (2017). *Impact evaluation of the prison-based Core Sex Offender Treatment Programme*. Ministry of Justice Analytical Series.

* Nicholaichuk, T. P. (1996). Sex Offender Treatment Priority: An Illustration of the Risk/Need Principle. *Forum on Corrections Research*, *8*(2), 38–41.

* Olver, M. E., Marshall, L. E., Marshall, W. L., & Nicholaichuk, T. P. (2020). A Long-Term Outcome Assessment of the Effects on Subsequent Reoffense Rates of a Prison-Based CBT/RNR Sex Offender Treatment Program With Strength-Based Elements. *Sexual Abuse*, *32*(2), 127–153. https://doi.org/10.1177/1079063218807486

* Ortmann, R. (2002). *Sozialtherapie im Strafvollzug: Eine experimentelle Längsschnittstudie zu den Wirkungen von Strafvollzugsmaßnahmen auf Legal- und Sozialbewährung*. Max-Planck-Institute for Foreign; International Criminal Law.

* Procter, E. (1996). A five year outcome evaluation of a community-based treatment programme for convicted sexual offenders run by the probation service. *Journal of Sexual Aggression*, *2*(1), 3–16. https://doi.org/10.1080/13552609608413253

* Rice, M. E., Quinsey, V. L., & Harris, G. T. (1991). Sexual recidivism among child molesters released from a maximum security psychiatric institution. *Journal of Consulting and Clinical Psychology*, *59*(3), 381–386. https://doi.org/10.1037//0022-006x.59.3.381

* Robinson, D. (1995). *The impact of cognitive skills training on post-release recidivism among Canadian federal offenders (User Report R-41)*. Correctional Service Canada.

* Romero, J. J., & Williams, L. (1983). Group psychotherapy and intensive probation supervision with sex offenders. *Federal Probation*, *47*, 36–42.

* Ruddijs, F., & Timmerman, H. (2000). The Stichting Ambulante Preventie ProjectenMethod: A Comparative Study of Recidivism in First Offenders in a Dutch Outpatient Setting. *International Journal of Offender Therapy and Comparative Criminology*, *44*, 725–739. https://doi.org/10.1177/0306624X00446008

* Schmid, P. (1989). *Was geschieht mit den Sexualstraftätern in der Psychiatrie? Darstellung und Bewertung der psychiatrischen Behandlung von Sexualstraftätern im Psychiatrischen Landeskrankenhaus Bad Schussenried in den Jahren 1978 - 1987*. na. https://books.google.ch/books?id=SElAvgAACAAJ

Schmucker, M., & Lösel, F. (2017). Sexual offender treatment for reducing recidivism among convicted sex offenders: a systematic review and meta-analysis. *Campbell Systematic Reviews*, *13*(1), 1–75. https://doi.org/https://doi.org/10.4073/csr.2017.8

* Smallbone, S. W., & McHugh, M. (2010). *Outcomes of Queensland Corrective Services Sexual Offender Treatment Programs. Final Report*. Queensland Government.

* Smid, W. J., Kamphuis, J. H., Wever, E. C., & Van Beek, D. J. (2016). A Quasi-Experimental Evaluation of High-Intensity Inpatient Sex Offender Treatment in the Netherlands. *Sexual Abuse*, *28*(5), 469–485. https://doi.org/10.1177/1079063214535817

* Taylor, R. (2000). *A seven-year reconviction study of HMP Grendon therapeutic community*. Development; Statistics Directorate Research Findings.

Team, R. C. (2022). *R: A language and environment for statistical computing*. R Foundation for Statistical Computing. https://www.R-project.org/

Viechtbauer, W. (2021). *Package "metafor": A Meta-Analysis Package for R*.

Worling, J. R., & Curwen, T. (2000). Adolescent sexual offender recidivism: success of specialized treatment and implications for risk prediction. *Child Abuse Negl*, *24*(7), 965–982. https://doi.org/10.1016/s0145-2134(00)00147-2

* Worling, J. R., Litteljohn, A., & Bookalam, D. (2010). 20-year prospective follow-up study of specialized treatment for adolescents who offended sexually. *Behavioral Sciences & the Law*, *28*(1), 46–57. https://doi.org/10.1002/bsl.912

* Ziethen, F. (2002). *Rückfallpräventive Effizienz der sozialtherapeutischen Behandlung von Sexualstraftätern. Evaluation der Sozialtherapie in der JVA Berlin-Tegel*.
